# Aberrant uterine folding in mice disrupts implantation chamber formation and alignment of embryo-uterine axes

**DOI:** 10.1242/dev.200300

**Published:** 2022-06-13

**Authors:** Manoj K. Madhavan, Francesco J. DeMayo, John P. Lydon, Niraj R. Joshi, Asgerally T. Fazleabas, Ripla Arora

**Affiliations:** 1 Department of Biomedical Engineering, Michigan State University, East Lansing, MI 48824, USA; 2 Institute for Quantitative Health Science and Engineering, Michigan State University, East Lansing, MI 48824, USA; 3 Reproductive and Developmental Biology Laboratory, National Institute of Environmental Health Sciences, Research Triangle Park, NC 27709, USA; 4 Department of Molecular and Cell Biology, Baylor College of Medicine, Houston, TX 77030, USA; 5 Department of Obstetrics, Gynecology and Reproductive Biology, Michigan State University, Grand Rapids, MI 49503, USA

**Keywords:** Uterine folds, Implantation chamber, Embryo-uterine orientation, Embryo morphogenesis, WNT5A, RBPJ

## Abstract

The uterine luminal epithelium folds characteristically in mammals, including humans, horses and rodents. Improper uterine folding in horses results in pregnancy failure, but the precise function of folds remains unknown. Here, we uncover dynamic changes in the 3D uterine folding pattern during early pregnancy with the entire lumen forming pre-implantation transverse folds along the mesometrial-antimesometrial axis. Using a time course, we show that transverse folds are formed before embryo spacing, whereas implantation chambers form as the embryo begins attachment. Thus, folds and chambers are two distinct structures. Transverse folds resolve to form a flat implantation region, after which an embryo arrives at its center to attach and form the post-implantation chamber. Our data also suggest that the implantation chamber facilitates embryo rotation and its alignment along the uterine mesometrial-antimesometrial axis. Using WNT5A- and RBPJ-deficient mice that display aberrant folds, we show that embryos trapped in longitudinal folds display misalignment of the embryo-uterine axes, abnormal chamber formation and defective post-implantation morphogenesis. These mouse models with disrupted uterine folding provide an opportunity to understand uterine structure-based mechanisms that are crucial for implantation and pregnancy success.

This article has an associated ‘The people behind the papers’ interview.

## Abstract (precis)

**Summary:** Uterine folds and implantation chambers are distinct structures and longitudinal folds in the uterine lumen disrupt implantation chamber formation, embryo-uterine axes alignment and implantation outcomes.

## INTRODUCTION

During early pregnancy, the uterus in mammals undergoes dynamic remodeling, guided by cellular and molecular events, to prepare for embryo implantation (Arora et al., 2016; Yuan et al., 2018). Changes in luminal epithelium (LE) morphology during pregnancy are crucial for embryo implantation and pregnancy outcomes (Cha et al., 2014; Daikoku et al., 2011; Tu et al., 2016; Zhang et al., 2014). In several mammals, including monotocous species such as humans, horses and cows, and polytocous species such as mice, rats, pigs and rabbits, the LE undergoes architectural changes to form structures called uterine folds (hereafter referred to as ‘luminal folds’) (Abd-Elkareem, 2017; Arora et al., 2016; Enders, 1975; Fissore et al., 1986; Jokubkiene et al., 2015). In mice, based on 2D histological sections, luminal folds are also referred to as crypts or regularly spaced luminal epithelial evaginations that extend from the primary lumen towards the anti-mesometrial (AM) pole on gestational day (GD) 3 of pregnancy (Cha et al., 2014; Daikoku et al., 2011). More recently, using confocal imaging and 3D reconstruction of the mouse uterus, we have shown that 2D crypts coincide with 3D luminal folds, suggesting that they are the same structure (Arora et al., 2016). The 3D luminal folding pattern changes significantly from a non-pregnant state to pregnant state, with folds running along the mesometrial-antimesometrial (M-AM) axis of the mouse uterus on GD3 (Arora et al., 2016). In humans, the earliest evidence of uterine (endometrial) folds dates back to 1973, when longitudinal folds were discovered in the uterine cavity using hysterography in both the proliferative and secretory phase of the menstrual cycle (Slezak and Tillinger, 1973). In 1994, Goldstein discovered folds in the endometrium using ultrasonohysterography, and called them ‘endometrial moguls’ (Goldstein, 1994). Recently, using saline contrast sonohysterography ∼50% women displayed endometrial folds in the secretory phase irrespective of uterine pathology (Jokubkiene et al., 2015). Owing to limitations of current technologies and ethical concerns associated with research in pregnant women, how endometrial folds form during the window of implantation and their function in pregnancy remain unknown.

A second structure formed by the LE in conjunction with the embryo is the implantation chamber (hereafter referred to as a ‘chamber’). In several species, including mice, rats, dogs and horses, the LE forms a chamber that holds the implanting embryo (Barrau et al., 1975; Enders and Liu, 1991; Enders, 1975). The structure of the chamber was first described in rats as a parabolic depression of the LE at the site of implantation (Enders, 1975). In mice, this chamber is a V-shaped structure containing the embryo (Arora et al., 2016; Enders, 1975; Yuan et al., 2018). Although, the terms ‘uterine crypts’ and ‘implantation chambers’ are often used interchangeably (Cha et al., 2014; Yuan et al., 2016, 2018), there is lack of a clear distinction between a crypt (fold) and a chamber. Thus, whether pre-implantation folds transform into post-implantation chambers is not known.

The region of the embryo that initiates attachment to the uterine lumen can differ between mammals. In women and horses, the polar trophectoderm of the embryo (embryonic pole) initiates attachment to the uterine LE (Ginther, 1983; Kirby et al., 1967). On the other hand, in rodents, the embryo aligns with the inner cell mass (ICM) and its polar trophectoderm towards the uterine M pole, and attachment initiates at the mural trophectoderm (abembryonic pole) that faces the uterine AM pole. Alignment of the embryo-uterine (E-U) axes is crucial for mammalian pregnancy. In horses, factors including uterine contractions and differential thickening of the uterine walls enable embryo orientation to place its embryonic pole at the uterine AM pole (Ginther, 1983; Silva and Ginther, 2006). However, the morphological features of the uterine lumen that enables E-U alignment during implantation remain to be discovered. Moreover, whether luminal folding pattern and chamber formation help with E-U alignment remains obscure.

Recently, using embryo location analysis, we have shown that embryo movement in the mouse uterus along the ovary-cervix (O-Cx) axis has three distinct phases: embryo entry (GD3 0000 h onwards); unidirectional clustered phase (GD3 0600 h onwards); and bidirectional spacing or scattering phase (GD3 1200 h onwards) (Flores et al., 2020). How the luminal folding pattern changes with embryo location is not known. We also showed that the number of implantation sites in the mouse uterus is not predetermined but rather depends on the number of embryos in the uterus, and that embryos implant at equidistant positions along the O-Cx axis (Flores et al., 2020). Whether potential implantation sites are established prior to or after the arrival of the embryo at the implantation site and whether the structure of the uterine lumen helps in equal spacing of implantation sites is not known.

In mice, based on 2D histological sections, abnormal LE histology during implantation has been linked to mid-gestation lethality and poor pregnancy outcomes (Cha et al., 2014; Tu et al., 2016; Zhang et al., 2014). In particular, WNT5A, a ligand in the non-canonical Wnt signaling pathway, and RBPJ, a mediator of the Notch signaling pathway, are both crucial for LE morphology and E-U alignment in the mouse uterus (Cha et al., 2014; Zhang et al., 2014). Moreover, embryo loss in WNT5A- and RBPJ-deficient mice has been attributed to abnormal LE morphology at the time of implantation. We recently reported that WNT5A-deficient mice also display an abnormal 3D luminal folding pattern. Although both WNT5A- and RBPJ-deficient mice display aberrations in LE, it is unclear how 3D luminal folding affects implantation, chamber formation and E-U alignment.

Here, we detail how dynamic changes in uterine luminal folding pattern facilitate implantation region formation, chamber formation and E-U alignment. Furthermore, we clarify the relationship between pre-implantation folds and post-implantation chambers, and how folds affect E-U alignment. Using WNT5A- and RBPJ-deficient mouse models, we show that aberrant folding pattern causes embryo trapping in folds, leading to disrupted E-U alignment, abnormal chamber formation and defective embryo morphogenesis that ultimately lead to embryo demise.

## RESULTS

### Uterine luminal folding dynamically changes along with uterine embryo location

**Fig. 1.**
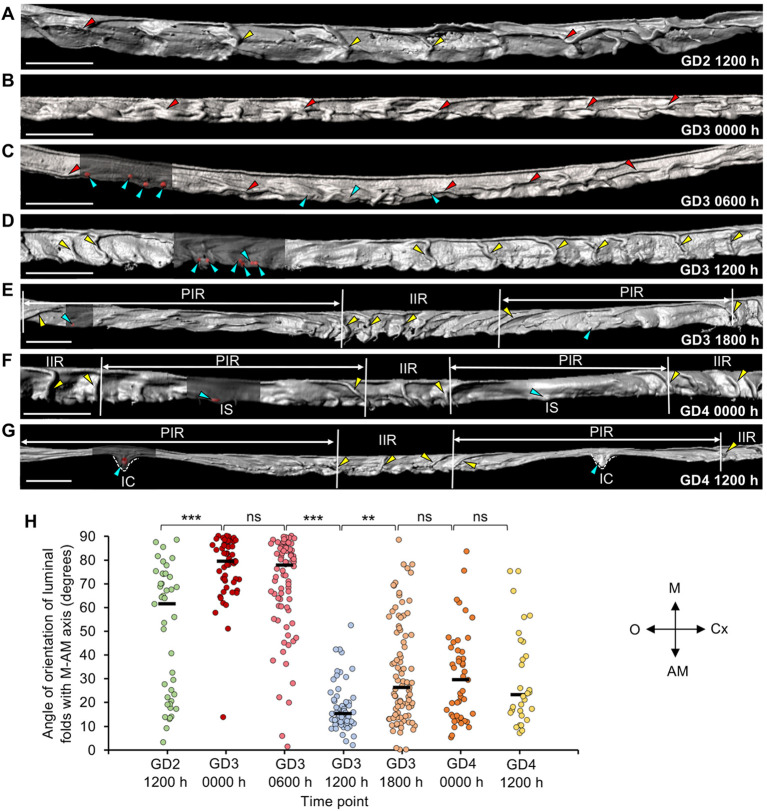
**Time course of luminal folding pattern from GD2 to GD4.** (A-G) 3D reconstruction of uterine lumen (gray) on (A) GD2 1200 h, (B) GD3 0000 h, (C) GD3 0600 h, (D) GD3 1200 h, (E) GD3 1800 h, (F) GD4 0000 h and (G) GD4 1200 h. Longitudinal folds disappear and the entire lumen has transverse folds along the M-AM axis at GD3 1200 h. Segments of lumen surfaces in C-G are made transparent to show embryos (red surfaces, blue arrowheads). Yellow arrowheads, transverse folds; red arrowheads, longitudinal folds. White dotted lines indicate implantation chamber. PIR, peri-implantation region; IIR, inter-implantation region; IC, implantation chamber; IS, implantation site; M, mesometrial pole; AM, anti-mesometrial pole; O, ovary; Cx, cervix. Scale bars: 1000 µm. (H) Quantification of fold orientation with respect to the M-AM axis in A-G. Black lines indicate the median angle. *n*=3 or 4 mice per time point. (*P*<0.001, Kruskal–Wallis test, Dunn's multiple comparison). **P<0.01, ***P<0.001; ns, non-significant.

To understand the relationship between luminal folding and embryo location, we evaluated uterine structure between noon on gestational day (GD) 2 (GD2 1200 h) and noon on GD4 (GD4 1200 h) (Fig. 1A-G and Movie 1) and quantified the angle between the luminal fold and the uterine M-AM axis (Fig. S1). At GD2 1200 h, when the embryos are in the oviduct (Flores et al., 2020), the luminal folds are present along both the M-AM axis (transverse folds) and O-Cx axis (longitudinal folds) (median=61.4°) (Fig. 1A,H). At GD3 0000 h, when clusters of embryos are present near the oviductal-uterine junction (Flores et al., 2020), the luminal folding pattern changes significantly and majority of folds are longitudinal (median=79.25°, *P*<0.0001, GD2 1200 h versus GD3 0000 h) (Fig. 1B,H, Fig. S2A and Movie 2). During clustered embryo movement, at GD3 0600 h (Flores et al., 2020), the majority of the folds continue to be longitudinal and embryos can be found in these folds (median=77.65°, *P*=0.96, GD3 0000 h versus GD3 0600 h) (Fig. 1C,H). Strikingly, at GD3 1200 h, prior to bidirectional scattering of embryos, the entire length of the lumen has only transverse folds along the M-AM axis (median=15.1°, *P*<0.0001, GD3 0600 h versus GD3 1200 h) (Fig. 1D,H and Movie 1). Notably, only ∼35% of embryos (11/31 embryos, *n*=4 mice) are found in these transverse folds, while the remaining ∼65% embryos are present in the flat regions in between the two folds (Fig. S2B). At GD3 1800 h, after embryo scattering, long stretches of flat luminal regions called peri-implantation regions (PIRs) begin to form. The regions between two PIRs, called inter-implantation regions (IIRs), retain the transverse folds (median=26.1°) (Fig. 1E,H). At GD4 0000 h, at the initiation of embryo implantation, the embryos are now present within the flat PIRs at the presumptive implantation sites (Fig. 1F, Figs S2C and S3). At GD4 1200 h, after embryo attachment, a V-shaped chamber forms at the implantation site in the middle of the PIR (Fig. 1G). Markedly, at this time, the folds in the IIRs continue to be aligned along the M-AM axis (median=23.1°) and there is no significant difference compared with GD4 0000 h (*P*>0.99) (Fig. 1H).

### The peri-implantation region is formed by resolution of transverse folds and stretching of the lumen along the O-Cx axis

**Fig. 2.**
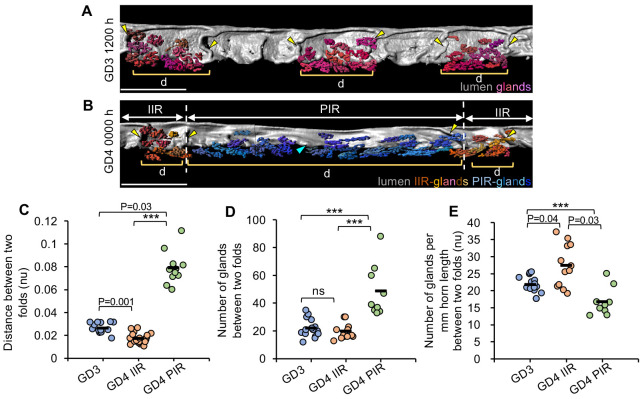
**Transverse folds along the M-AM axis resolve to form peri-implantation regions.** (A,B) 3D surfaces of uterine lumen and glands at GD3 1200 h (A) and GD4 0000 h (B). In A, gland surfaces are displayed for alternate regions for easy visualization. PIR, peri-implantation region; IIR, inter-implantation region; d, region between two folds. Scale bars: 1000 µm. Yellow arrowheads indicate transverse folds; blue arrowheads indicate embryos; orange indicates pseudocolored glands in IIR; blue indicates pseudocolored glands in PIR. (C-E) At GD3 1200 h, GD4 0000 h IIRs and GD4 0000 h PIR, quantification of the distance between two consecutive transverse luminal folds (C), quantification of the number of glands between two luminal folds (D) and quantification of the number of glands between two folds per mm of PIR horn length (E). *n*=3 mice per group. (***P<0.001, Kruskal–Wallis test, Dunn's multiple comparison)**.** nu, normalized units. Black horizontal lines indicate the mean value.

To discern the relationship between folds at GD3 and GD4, we first evaluated the distance between two consecutive transverse folds along the M-AM axis in three groups: GD3 1200 h (GD3), GD4 0000 h IIRs (GD4 IIRs) and GD4 0000 h PIRs (GD4 PIRs) (Fig. 2A,B and Fig. S4). The distances are normalized to the length of the uterine horn to account for the variation among mice and are represented as normalized units (nu). We found a significant difference between the three groups (*P*<0.001, Kruskal–Wallis test, Dunn's multiple comparison). The mean distance between two folds at GD4 PIRs (mean=0.079nu) is significantly higher compared with both GD3 (mean=0.026nu, *P*=0.03) and GD4 IIRs (mean=0.017 nu, *P*<0.001) (Fig. 2C). We also observe that the mean distance between folds at GD4 IIRs is significantly lower than at GD3 (*P*<0.001). As there is no epithelial proliferation beyond GD3 (Haraguchi et al., 2014; Hiraoka et al., 2020), increased length of the PIR at GD4 could be due to a stretching mechanism or resolution (flattening) of transverse folds formed at GD3. To distinguish between these two possibilities, we quantified the number of glands between two folds in the three groups. We predicted that if the primary mechanism of PIR formation is resolution of folds, then the number of glands between two folds at GD4 PIRs should be higher than the number of glands between two folds at GD4 IIRs and between two folds at GD3. Indeed, the average number of glands between two folds at GD4 PIRs (mean=48.6 glands) is approximately twofold higher compared with both GD3 (mean=22 glands, *P*<0.001) and GD4 IIRs (mean=19.5 glands, *P*<0.001) (Fig. 2D). In addition, there is no significant difference between the total number of glands per unit length of horn at GD3 1200 h and GD4 0000 h (PIRs and IIRs combined) (*P*=0.38, Mann–Whitney *U*-test) (Fig. S5). Taken together, these data support resolution of folds to form PIRs. Furthermore, when normalized for horn length, we observed that the average number of glands per millimeter (mm) of horn length at GD4 PIRs (mean=16.8 glands/mm) is lower compared with both GD3 (mean=21.74 glands/mm, *P*<0.001) and GD4 IIRs (mean=27.45 glands/mm, *P*=0.03) (*P*<0.001, Kruskal–Wallis test, Dunn's multiple comparison) (Fig. 2E). The decrease in the number of glands per mm at PIRs can be due to some stretching of the lumen to resolve folds or due to the added length from the resolved folds. However, we also observed an increase in the average number of glands per mm at GD4 IIRs compared with GD3 (*P*=0.04), although the number of glands between two consecutive folds in these two groups is the same (Fig. 2E). These data suggest that the lumen in the IIRs is likely compressed, which would support a stretching mechanism for the lumen to form PIRs. Overall, our results suggest that PIRs primarily form as a result of resolution of transverse folds; however, lumen stretching along the O-Cx axis also contributes to PIR formation.

### Luminal patterning to form flat peri-implantation regions precedes fine embryo spacing

**Fig. 3.**
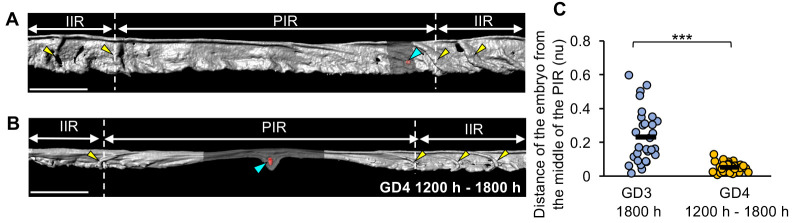
**The peri-implantation region is formed prior to arrival of the embryo at the implantation site.** (A,B) 3D surface of uterine lumen and embryo at GD3 1800 h (A) and GD4 1200 h (B). Yellow arrowheads indicate transverse folds. Blue arrowheads indicate embryos (red surfaces). Scale bars: 1000 µm. At GD3 1800 h, the embryo is present near the PIR margin closer to the transverse folds in the IIR. At GD4, the embryo is present near the PIR center. (C) Quantification of the distance of embryos from the center of PIR at GD3 1800 h (*n*=7 mice, number of PIRs=29) and GD4 1200 h to 1800 h (*n*=6 mice, number of PIRs=29). Distances are normalized to PIR length. Black lines indicate the mean distance. ***P<0.001, Mann–Whitney *U*-test. PIR, peri-implantation region; IIR, inter-implantation region. nu, normalized units.

Whether the resolution of transverse folds to form PIRs occurs prior to or after the arrival of the embryo at the implantation site is not known. At GD3 1800 h, when PIRs are first observed, embryos are commonly present at the margins of the flat PIRs, closer to the IIR fold on either end (Fig. 3A). Post-implantation, the embryo is always present closer to the middle of the PIR (Fig. 3B). We quantified this by measuring the distance of an embryo from the middle of the PIR at GD3 1800 h and post-implantation between GD4 1200 h and GD4 1800 h. The distances are normalized to the length of the PIR. We observe that the mean distance of the embryos from the middle of the PIRs on GD3 1800 h (mean=0.23nu) is significantly higher than the post-implantation time points (mean=0.05nu) (*P*<0.001, Mann–Whitney *U*-test) (Fig. 3C).

Previously, we have shown that the glands in the inter-implantation region reorient towards the implantation site at GD4 1200 h (Arora et al., 2016). The site of embryo implantation always coincides with the center of the gland reorientation site. Here, we show additional evidence that gland orientation occurs as early as GD3 1800 h, when PIRs are first observed (Fig. S6A,A′). Interestingly, the center of the gland reorientation site coincides with the center of PIRs at GD3 1800 h (Fig. S6A,A′). However, the embryos at GD3 1800 h are not found at the gland reorientation sites, whereas at GD4 1200 h, the implantation site, the center of the gland reorientation site and the PIR coincide (Fig. S6B,B′). Hence, glands reorient towards the center of the PIRs even before embryos arrive at the potential implantation site. These data support that the PIR and gland reoriented sites are formed prior to embryo arrival at the implantation site.

### Embryos localize in aberrant longitudinal folds instead of flat peri-implantation regions in *Wnt5a^cKO^* uteri

**Fig. 4.**
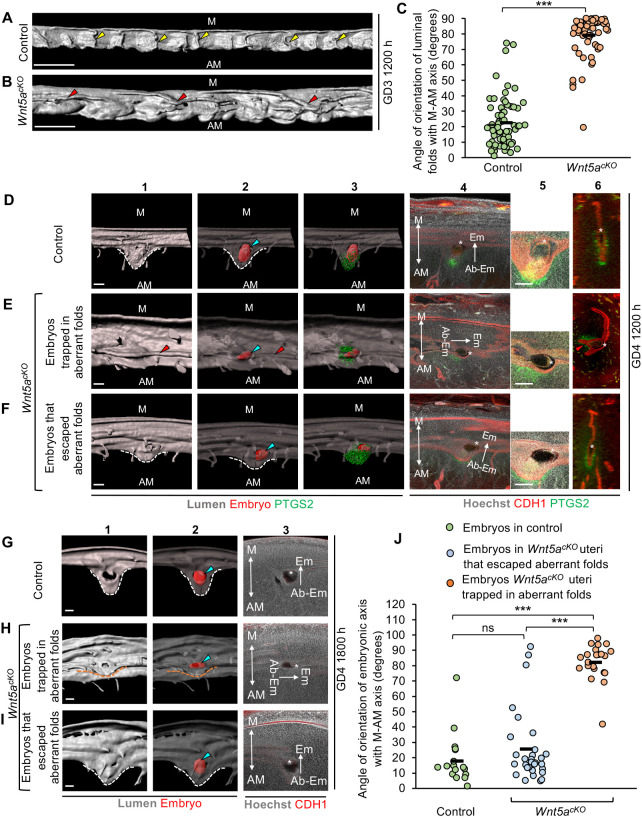
**Aberrant pre-implantation folding in *Wnt5a^cKO^* leads to disrupted embryo-uterine axes alignment and abnormal chamber formation.** (A,B) 3D reconstruction of lumen in control (A) and *Wnt5a^cKO^* (B) uteri at GD3 1200 h. Yellow arrowheads indicate transverse folds. Red arrowheads indicate longitudinal folds. Blue arrowheads indicate embryos (red surface). Scale bars: 1000 µm. (C) Quantification of fold angle with M-AM axis in control and *Wnt5a^cKO^* (*n*=3 mice/group) uteri on GD3 1200 h (***P<0.001, Mann–Whitney *U*-test). (D-F) 3D surface view and optical slice view of implantation sites at GD4 1200 h in control (D) and *Wnt5a^cKO^* (E,F) uteri. Implantation sites in *Wnt5a^cKO^* uteri where embryos are trapped in folds (E) or where embryos have escaped folds (F). Panel 1: 3D lumen surface (gray). Panel 2: transparent 3D lumen and embryo (red) surface. Panel 3: transparent 3D lumen, embryo and PTGS2 (green) surface. Panel 4: optical slice with frontal view. Panel 5: high-magnification image of embryos in panel 4. Panel 6: optical slice with transverse view. Scale bars: 100 µm. (G-I) 3D surface view and optical slice view of implantation sites on GD4 1800 h in control (G) and *Wnt5a^cKO^* (H,I) uteri. Implantation sites in *Wnt5a^cKO^* uteri where embryos are trapped in folds (H) or where embryos have escaped folds (I). Panel 1: 3D lumen surface (gray). Panel 2: transparent 3D lumen and embryo (red) surface. Panel 3: optical slice with frontal view. Scale bars: 100 µm. (J) Quantification of embryo orientation with respect to M-AM axis in control (*n*=3 mice, *n*_e_=26 embryos) and *Wnt5a^cKO^* (*n*=6 mice, *n*_e_=52 embryos) uteri at GD4 1800 h. (***P<0.001, Kruskal–Wallis test, Dunn's multiple comparison). ns, non-significant. M, mesometrial pole; AM, anti-mesometrial pole; Em, embryonic pole; Ab-Em, abembryonic pole. Black dashes indicate the mean angle. Asterisks in D-I indicate inner cell mass.

WNT5A-deficient mice display abnormal 2D luminal histology, leading to defective implantation, decidualization, embryo orientation and placentation, with around 67% of the embryos dying mid-gestation (Cha et al., 2014). Furthermore, these mice display longitudinal (and not transverse) folds during peri-implantation stages of pregnancy (Arora et al., 2016). To examine how aberrant folding disrupts implantation, we combined uterine-specific *Pgr-Cre* with a *Wnt5a* conditional allele to generate *Pgr^Cre/+^;Wnt5a^flox/flox^* (*Wnt5a^cKO^*) mice*.* Notably, our studies in Fig. 1 were performed with CD1 (mixed-background) mice, whereas the *Wnt5a^cKO^* mice were on the C57BL/6 background. First, we observed that, at GD3 1200 h, control uteri (*Wnt5a^flox/flox^*) displayed only transverse folds (mean=22.56°) (Fig. 4A,C). These data suggest that uterine folding occurs independently of mouse genetic background. The *Wnt5a^cKO^* uteri have aberrant longitudinal folds aligned along the O-Cx axis (mean=79.08°) and are significantly different compared with controls (*P*<0.001, Mann–Whitney *U*-test) (Fig. 4B,C and Fig. S7). In addition, we noted that the width of the lumen along M-AM axis in the *Wnt5a^cKO^* was significantly higher compared with controls (*P*=0.01, Mann–Whitney *U*-test) (Fig. S8). During implantation at GD4 1200 h, in the control uteri, 100% of embryos (14 embryos, *n*=3 mice) implant in the flat PIR (Fig. 4D). However, in *Wnt5a^cKO^* uteri at GD4 1200 h, aberrant longitudinal folds persist in PIRs and ∼37% of embryos (6/16 embryos, *n*=3 mice) are trapped in these longitudinal folds away from the AM-pole (Fig. 4E).

### Embryos in longitudinal folds display defective embryo-uterine axes alignment and chamber formation

At GD4 1200 h in the control uteri, embryos attach with their mural trophectoderm facing the AM pole (Fig. 4D). Additionally, PTGS2 (COX2), a marker for decidualization, is expressed in the subepithelial stroma under the mural trophectoderm at the chamber AM pole. Embryos trapped in folds in the *Wnt5a^cKO^* uteri do express PTGS2 at the mural trophectoderm side of the embryo, but this expression is not at the AM pole. This suggests a misalignment of the embryo axis along the uterine axis. Moreover, implantation sites with embryos trapped in longitudinal folds fail to initiate chamber formation at GD4 1200 h (Fig. 4E). Embryos in *Wnt5a^cKO^* uteri that escape the longitudinal folds attach in flat regions at the AM pole and form V-shaped chambers (Fig. 4F).

*Wnt5a^cKO^* mice display delayed implantation (Cha et al., 2014) and, thus, at GD4 1200 h embryos could display delayed chamber formation and E-U alignment, even if they are not trapped in longitudinal folds. Thus, we performed implantation site analysis and embryo-uterine axes angle quantification, at GD4 1800 h (Figs S9A and S10A,A′). In the control uteri, 100% of the embryos are located in a V-shaped chamber at the AM pole and over 96% of the embryos have their embryonic-abembryonic (Em-AbEm) axis aligned with the uterine M-AM axis (mean=17.8°) (Fig. 4G,J and Table 1). On the other hand, in *Wnt5a^cKO^* uteri at GD4 1800 h, we observe two groups of embryos. Around 42% of the embryos are trapped in aberrant longitudinal folds and over 95% of those (40.38% of total) have disrupted E-U alignment. Instead of the Em-AbEm axis being parallel to the M-AM axis, 95% of the embryos trapped in folds have their axis almost perpendicular to the M-AM axis (mean=82.22°, *P*<0.001) (Fig. 4H,J and Table 1). Moreover, embryos trapped in folds have smaller chambers growing away from the AM pole, along the left-right axis of the uterus. The remaining 58% of embryos in the *Wnt5a^cKO^* uteri that have escaped the aberrant longitudinal folds, appear similar to the control uteri with a V-shaped chamber at the AM pole and over 90% of those have their Em-AbEm axis aligned along the M-AM axis (mean=25.48°, *P*>0.05) (Fig. 4I,J and Table 1).

**Table 1.**
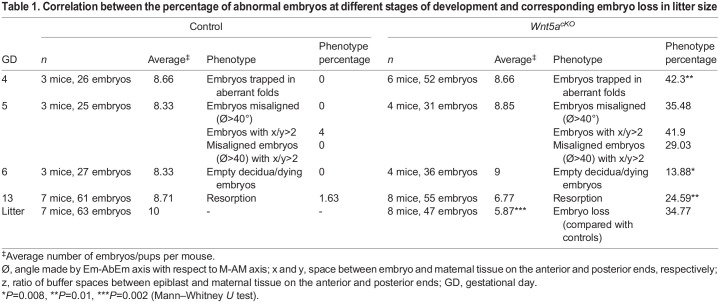
Correlation between the percentage of abnormal embryos at different stages of development and corresponding embryo loss in litter size

### Implantation chamber formation mediates embryo-uterine axes alignment

**Fig. 5.**
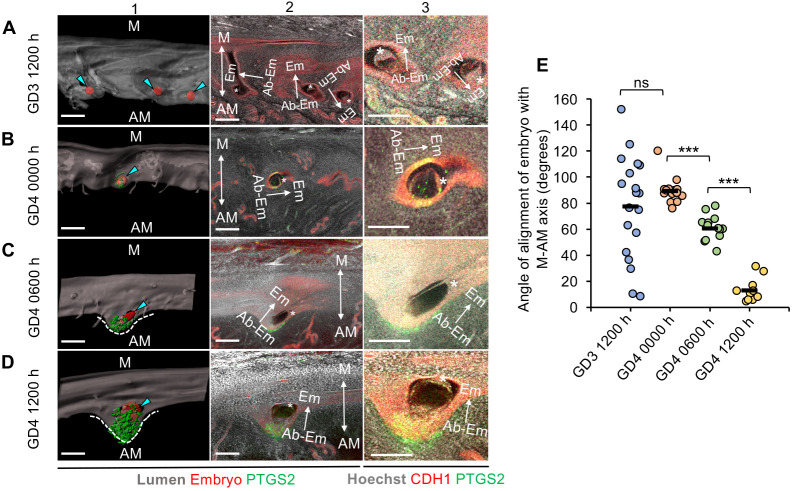
**Alignment of embryo-uterine axes is facilitated by the formation and elongation of the chamber.** (A-D) The relationship between chamber formation and embryo orientation with respect to M-AM axis in control mice at GD3 1200 h (A), GD4 0000 h (B), GD4 0600 h (C) and GD4 1200 h (D). Panel 1: transparent lumen with PTGS2 (green) and embryo (red). Panel 2: optical slices with CDH1 (red), PTGS2 (green) and Hoechst (gray). Panel 3: high-magnification images of embryos in panel 2. At GD4 0600 h, the embryo appears to be orienting its ICM towards the M pole but is still in contact with one wall of the chamber. Blue arrowheads indicate embryos (red surfaces), White dotted lines indicate the implantation chamber. Asterisks indicate the ICM. M, mesometrial pole; AM, anti-mesometrial pole. Scale bars: 100 µm. (E) Quantification of embryo orientation with respect to the M-AM axis at GD3 1200 h (*n*=3, *n*_e_=19), GD4 0000 h (*n*=3, *n*_e_=15), GD4 0600 h (*n*=4, *n*_e_=14) and GD4 1200 h (*n*=4, *n*_e_=11). *n*, number of mice; *n*_e_, number of embryos. Black lines indicate the mean angle. ***P<0.001; ns, non-significant; Mann–Whitney *U* test.

Although we observe that in the *Wnt5a^cKO^* uteri, 95% of embryos trapped in longitudinal folds have disrupted E-U alignment, the mechanism by which the embryos align with the uterine M-AM axis remains unclear. To understand how the lumen structure and the folding pattern facilitate alignment of the embryo-uterine axes, we examined embryo orientation relative to the luminal structure in control mice from GD3 1200 h to GD4 1200 h. At GD3 1200 h, during the clustered phase of embryo movement (Flores et al., 2020), the Em-AbEm axis of the embryos is randomly oriented with respect to the M-AM axis [mean=77.57±39.17° (s.d.)] (Fig. 5A,E and Fig. S11A). At the onset of implantation, at GD4 0000 h, the Em-AbEm axis of the embryos is almost perpendicular to the M-AM axis (mean=89.58±9.87°) (Fig. 5B,E). The initiation of implantation is evident from the expression of PTGS2 in the LE, near the mural trophectoderm at the abembryonic pole of the embryo (Fig. 5B and Fig. S11B) (Scherle et al., 2000). A few hours later, at GD4 0600 h, a small chamber is formed at the AM pole and expression of PTGS2 shifts from the LE to the stroma under the chamber. Concurrent with chamber formation, embryo rotation is also initiated with the embryos oriented at an acute angle with respect to the M-AM axis (mean=60.7±17.54°, *P*<0.001, GD4 0000 h versus GD4 0600 h) (Fig. 5C,E and Fig. S11C). Notably, one side of the embryo is in contact with the wall of the chamber in 86% of embryos (*n*=12/14 embryos from three mice). This suggests that the embryo may depend on the chamber for rotation. At GD4 1200 h, consistent with the increase in the size of the chamber at the AM pole, embryo rotation is complete with its axis almost parallel to the uterine M-AM axis (mean=13.10±9.07°, *P*<0.001, GD4 0600 h versus GD4 1200 h) (Fig. 5D,E and Fig. S11D).

We postulated that if embryo rotation is dependent on the implantation chamber, then during the period when rotation is observed (between GD4 0600 h and GD4 1200 h), the position of the embryo with respect to the chamber should stay constant. To this end, we used the ICM (for embryo position) and assessed its location with respect to the expression of stromal PTGS2 under the chamber (for chamber position). We measured the angle between the embryonic-abembryonic axis and the embryonic (ICM)-PTGS2 axis at GD4 0600 h and GD4 1200 h (Fig. S12A,B). We observe that there is no significance difference in the mean angle between these two axes at GD4 0600 h (mean=8.2°) and at GD4 1200 h (mean=9.8°). This suggests that the relative position of the embryo and stromal PTGS2 under the chamber is maintained during embryo rotation (*P*=0.88, Mann–Whitney *U*-test) (Fig. S12C). These data suggest that embryo-uterine orientation during implantation is facilitated by formation of a chamber at the AM pole in flat PIRs.

Based on these data, we hypothesized that for axis alignment, embryos in the *Wnt5a^cKO^* uteri should behave similar to embryos in control uteri until chamber formation initiates. Consequently, at GD4 0000 h, we observed that, similar to controls (Fig. S13A), all embryos in the *Wnt5a^cKO^* (including 33% of embryos trapped in longitudinal folds) are aligned with their Em-AbEm axis almost perpendicular to the M-AM axis irrespective of their localization (Fig. S13B,C). Thus, in *Wnt5a^cKO^*, embryos in longitudinal folds stay perpendicular to the M-AM axis from GD4 0000 h until GD4 1800 h (Fig. 4H), while embryos that escape longitudinal folds are able to align along the M-AM axis at GD4 1800 h, concomitant with chamber formation (Fig. 4I).

### Misalignment of embryo-uterine axis in *Wnt5a^cKO^* uteri leads to defective post-implantation embryo morphogenesis

**Fig. 6.**
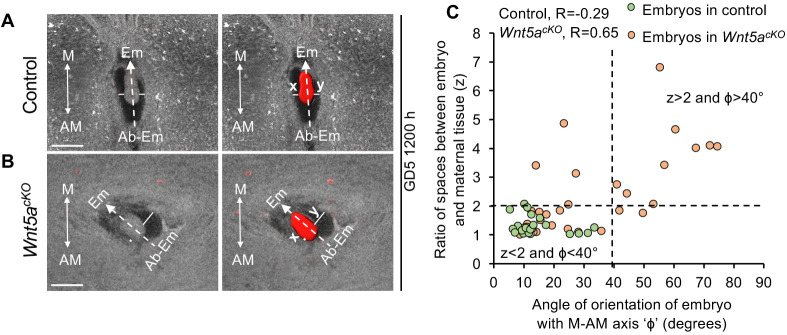
**Embryo-uterine misalignment leads to defective buffer space between epiblast and maternal decidua.** (A,B) 2D optical slices of implantation sites in control (A) and *Wnt5a^cKO^* (B) uteri at GD5 1200 h. 3D surface of the embryo (red, right panel). There is uneven buffer space between epiblast and maternal decidua in *Wnt5a^cKO^* uteri. Scale bars: 1000 µm. (C) Quantification of embryo orientation with respect to the M-AM axis, and ratio of buffer spaces between epiblast and maternal decidua at GD5 1200 h in controls (*n*=3, *n*_e_=25) and *Wnt5a^cKO^* (*n*=3, *n*_e_=33) uteri. *n*, number of mice; *n*_e_, number of embryos. (Pearson's correlation coefficient, R for control= −0.29, ns, *P*>0.05; R for *Wnt5a^cKO^*=0.65, ***P<0.001). z, maximum(x,y)/minimum(x,y). M, mesometrial pole; AM, anti-mesometrial pole; Em, embryonic pole; Ab-Em, abembryonic pole.

To determine the effect of embryo-uterine misalignment on embryo morphogenesis, we analyzed implantation sites within decidua in *Wnt5a^cKO^* uteri and control uteri at GD5 1200 h. We observe that, in the *Wnt5a^cKO^* uteri, embryos continue to be misaligned. We also observe that the space between the embryo and the maternal decidua is abnormal in the embryos that are misaligned. We measured the ratio of spaces between the maternal tissue and the epiblast of the embryo on the anterior and posterior ends (z), and the angle between the embryo axis and the M-AM axis (φ) (Hiramatsu et al., 2013). In control uteri, 100% of the embryos are oriented along the M-AM axis or proximal-distal axis (mean φ=14.26°, Table 1) and the value of z is closer to 1 (mean z=1.31) (Fig. 6A,C). However, in the *Wnt5a^cKO^* uteri, the mean φ is higher compared with the control uteri (mean φ=31.49°, *P*<0.001, Mann–Whitney *U*-test) (Fig. 6B,C). 35% of the embryos appear to be severely misaligned (φ>40°). Furthermore, the embryos have highly uneven buffer spaces (mean z=2.35, *P*<0.001, Mann–Whitney *U*-test) with ∼42% of the embryos displaying z>2. Strikingly, 81% of embryos (29% of total) that are misaligned along the uterine axis have z>2 (Table 1). Correlation analysis shows a significant correlation between E-U alignment angle and ratio of spaces between embryo and maternal tissue in *Wnt5a^cKO^* uteri (Pearson's correlation coefficient R=0.65, *P*<0.001). These data suggest that E-U misalignment leads to defective embryo morphogenesis.

### Defective embryo-uterine axes alignment in *Rbpj^cKO^* uteri is due to aberrant longitudinal folds

**Fig. 7.**
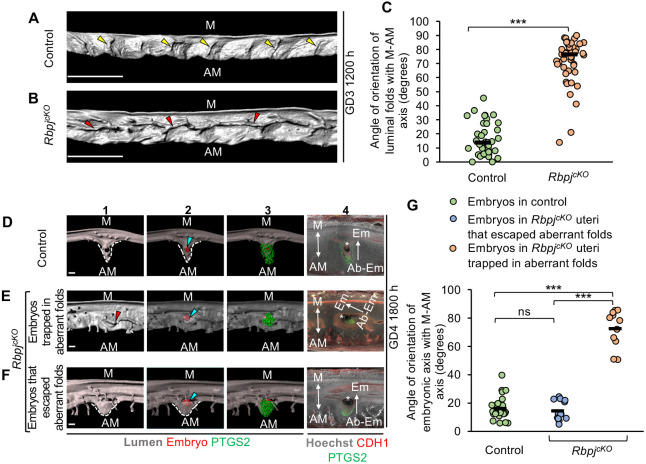
**Longitudinal folds in *Rbpj^cKO^* disrupt embryo-uterine axes alignment and chamber formation.** (A,B) 3D reconstruction of lumen in control (A) and *Rbpj^cKO^* (B) uteri on GD3 1200 h. Yellow arrowheads indicate transverse folds. Red arrowheads indicate longitudinal folds. Scale bars: 1000 µm. (C) Quantification of fold orientation with respect to the M-AM axis in control and *Rbpj^cKO^* (*n*=3 or 4 mice/group) uteri at GD3 1200 h (***P<0.001, Mann–Whitney *U* test). (D-F) 3D surface view and optical slice view of implantation sites at GD4 1800 h in control (D) and *Rbpj^cKO^* (E,F) uteri. Implantation sites in *Rbpj^cKO^* uteri where embryos are trapped in folds (E) or where embryos have escaped folds (F). Panel 1: 3D lumen surface (gray). Panel 2: transparent 3D lumen surface with embryo surface (red). Panel 3: transparent 3D lumen surface with embryo and PTGS2 (green) surface. Panel 4: optical slice with frontal view. Scale bars: 100 µm. Blue arrowheads indicate embryos (red surfaces). Asterisks indicate inner cell mass. (G) Quantification of embryo orientation with respect to the M-AM axis in control (*n*=3, *n*_e_=24) and *Rbpj^cKO^* (*n*=3, *n*_e_=24) uteri at GD4 1800 h. *n*, number of mice; *n*_e_, number of embryos. (***P<0.001, Kruskal–Wallis test, Dunn's multiple comparison). ns, non-significant. M, mesometrial pole; AM, anti-mesometrial pole; Em, embryonic pole; Ab-Em, abembryonic pole. Black lines in C,G indicate the mean angle.

E-U misalignment in *Wnt5a^cKO^* mice could be due to the effect of WNT5A signaling on uterine folding or due to a different process regulated by WNT5A. We hypothesized that mutants with aberrant folding pattern must show defective E-U alignment irrespective of the signaling pathway involved. Thus, we searched the literature for genetic mutants with known defects in E-U misalignment without a known effect on uterine folding. We examined RBPJ-deficient mice with known defects in E-U alignment and predicted that the E-U misalignment in these mice would be a result of aberrant pre-implantation folds. We generated RBPJ-deficient mice (*Rbpj^cKO^, Pgr^Cre/+^;Rbpj^flox/flox^*) by combining a *Rbpj* conditional allele (*Rbpj^flox/flox^*) with the *Pgr-Cre.* At GD3 1200 h, control uteri (*Rbpj^flox/flox^*) have transverse folds (mean=16.18°) (Fig. 7A,C). In contrast, as predicted, the folding pattern in *Rbpj^cKO^* uteri is aberrant, with longitudinal folds running along O-Cx axis instead of transverse folds along M-AM axis (mean=71.79°, *P*<0.001, Mann-Whitney *U*-test) (Fig. 7B,C). Post-implantation, at GD4 1800 h, embryos in control uteri are located in V-shaped chambers at the AM pole in flat PIRs, with their Em-AbEm axis aligned along the M-AM axis (mean=15.96°) (Fig. 7D,G). However, in *Rbpj^cKO^* uteri, similar to *Wnt5a^cKO^* uteri, we observe two groups of embryos. About 46% of embryos in *Rbpj^cKO^* uteri are trapped in aberrant longitudinal folds retained at PIRs, with abnormal expression pattern of PTGS2 and defective chamber formation. Furthermore, 100% of embryos trapped in folds have disrupted E-U alignment when compared with control uteri (mean=72.32°, *P*<0.001, Kruskal–Wallis test, Dunn's multiple comparison) (Fig. 7E,G). The remaining 54% of the embryos in the *Rbpj^cKO^* uteri that have escaped aberrant folds have a V-shaped chamber, normal PTGS2 expression pattern and normal E-U alignment, similar to control uteri (mean=14.35°, *P*>0.05, Kruskal–Wallis test, Dunn's multiple comparison) (Fig. 7F,G). Taken together, these results suggest that longitudinal folds in the pre-implantation uterus are detrimental to E-U alignment and proper chamber formation, irrespective of the signaling pathway affected.

## DISCUSSION

**Fig. 8.**
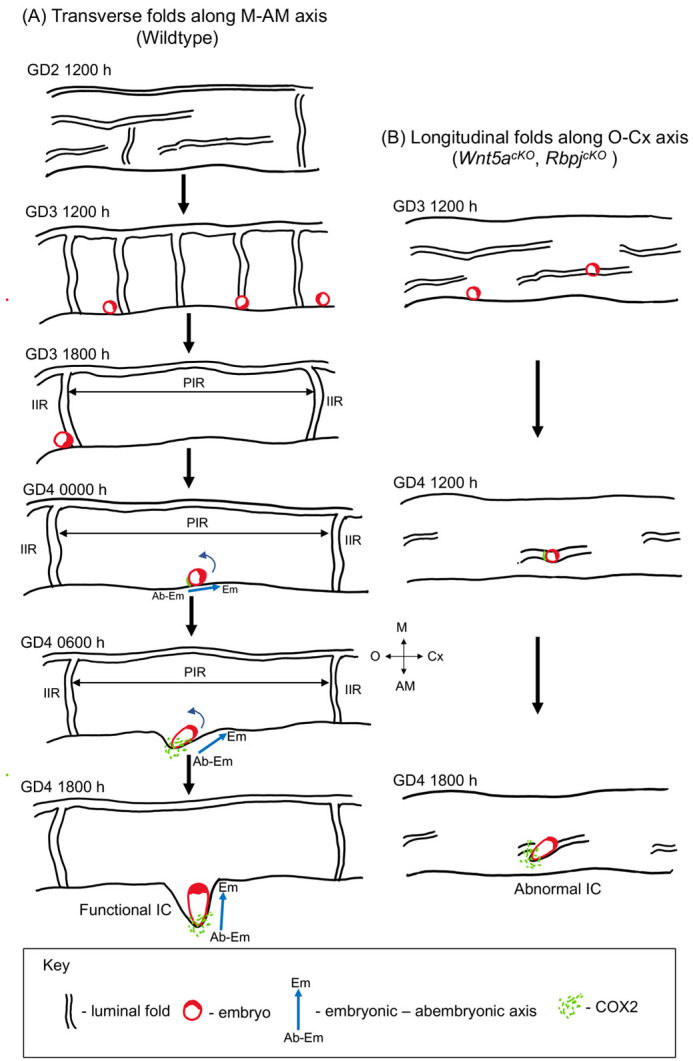
**Schematic showing the effect of uterine luminal folding pattern on embryo implantation, orientation and chamber formation.** (A) During mouse pregnancy, uterine lumen displays transverse folds along the M-AM axis at GD3 1200 h. These folds resolve in some regions to form flat PIRs at GD3 1800 h. PIRs are formed before the arrival of the embryo at the implantation site. Embryos arrive in the middle of the PIR, with their Em-AbEm axis perpendicular to the uterine M-AM axis at GD4 0000 h. Embryo attachment to the uterine luminal epithelium initiates at the mural trophectoderm of the embryo. Embryo orientation along the M-AM axis takes place as the implantation chamber forms and elongates towards the AM pole. (B) In models with aberrant uterine folding (*Wnt5a^cKO^* and *Rbpj^cKO^*), longitudinal folds aligned with the O-Cx axis are observed at GD3 1200 h. Folds along the O-Cx axis fail to resolve to form flat PIRs, resulting in embryos becoming trapped at GD4 1200 h. Embryos trapped in longitudinal folds display defective chamber formation and disrupted alignment of the embryo-uterine axes at GD4 1800 h. M, mesometrial pole; AM, anti-mesometrial pole; O, ovary; Cx, cervix; Em, embryonic pole; Ab-Em, abembryonic pole; IC, implantation chamber; IS, implantation site; PIR, peri-implantation region; IIR, inter-implantation region.

Our study delineates how the 3D structure of the uterine lumen during early pregnancy is crucial to embryo implantation, chamber formation, E-U alignment and embryo morphogenesis (Fig. 8). Our study shows that: (1) the randomly folded uterine lumen organizes into longitudinal folds during unidirectional embryo movement and later into transverse folds during embryo spacing prior to implantation; (2) transverse folds are resolved to form flat peri-implantation regions; (3) peri-implantation regions are pre-established by luminal patterning, prior to completion of embryo spacing; (4) luminal folds (or 2D crypts) and implantation chambers are distinct structures; (5) chamber formation facilitates E-U alignment; (6) aberrant longitudinal folds trap embryos, leading to E-U misalignment and abnormal chamber formation; and (7) mis-alignment of embryos leads to abnormal buffer space between epiblast and maternal tissue, likely contributing to poor embryo growth later in pregnancy.

### Uterine folds: differences and similarities between mice and other species

Knowledge pertaining to size and orientation of folds with respect to the uterine axis varies depending on the species and stage of estrous cycle, and is heavily dependent on the plane of examination (Abd-Elkareem, 2017; Enders and Liu, 1991; Fissore et al., 1986; Kähn et al., 1989; Slezak and Tillinger, 1973). This is because longitudinal folds can be visualized only when the tissue is sliced along a transverse plane and transverse folds can only be visualized when the tissue is examined along the longitudinal plane. Most of the conventional visualization methods, such as ultrasound and histology, are performed in the transverse plane and can easily detect longitudinal folds but often miss the transverse folds. Consequently, longitudinal folds along the long axis of the uterus have been observed in multiple species, including the human, rabbit, horse, donkey and bushbaby uterus (Ginther, 1983; Njogu et al., 2013; Renner-Martin et al., 2009). In humans, longitudinal folds have been observed during both the secretory and proliferative phases of the menstrual cycle but the orientation of folds during pregnancy is not known and requires future investigation (Jokubkiene et al., 2015; Slezak and Tillinger, 1973).

Ultrasound examination of the bovine uterus in the transverse plane revealed distinct longitudinal folds at the time of estrous; these folds disappeared at the time of diestrus or pregnancy (Fissore et al., 1986). However, in a different study, 3D examination using nuclear magnetic resonance imaging in the pregnant bovine uterus revealed crescent-shaped transverse folds that are perpendicular to the long axis of the uterine horn (Kähn et al., 1989). Thus, it is important to assess folding in different planes to fully understand the structural changes associated with the uterine lumen in preparation for pregnancy. In our study using mice, we observed both longitudinal folds (along O-Cx axis) and transverse folds (along M-AM axis) prior to embryo entry into the uterus and only transverse folds during pre-implantation phase of pregnancy. Our observations during mouse pregnancy are similar to those made in bovines where longitudinal folds disappear while transverse folds persist during pregnancy.

In rabbits, six longitudinal folds are observed after induction of ovulation during pseudopregnancy, and the complexity of the folding pattern changes with the stage of pseudopregnancy (Abd-Elkareem, 2017). We observe a similar change in the complexity of folding pattern in the mouse uterus, depending on the timing of pregnancy and embryo location. In horses, during ovulation, endometrial folds display a cartwheel appearance. Abnormally thick endometrial folds displaying a disrupted cartwheel pattern are indicative of endometritis and lead to poor pregnancy prognosis (Hayes et al., 1985; Samper, 2010). Similarly, in mice, we observe that disrupted folding pattern, as in the case of *Wnt5a^cKO^* and *Rbpj^cKO^* mice, leads to poor pregnancy outcomes. Although data on folding have been documented in other mammalian species, our data show for the first time that orientation of uterine folds with respect the uterine axis is important for the alignment of embryo to the uterine axis, opening doors for similar investigations in other species.

### Folds may be necessary for embryo movement but are dispensable for embryo spacing

Before embryo entry, both longitudinal and transverse folds are present in the mouse uterus, but only longitudinal folds remain during and immediately after embryo entry. Thus, we speculate that longitudinal folds aid in the transport of embryos from the oviductal end towards the center of the horn during unidirectional clustered embryo movement. These observations are similar to those in horses where longitudinal endometrial folds are believed to enable the movement of the embryo through the entire length of the uterus before the embryo implants (Ginther, 1985). Embryos in both *Wnt5a^cKO^* and *Rbpj^cKO^* uteri are trapped in longitudinal folds along O-Cx axis, away from the AM pole, thus transverse folds along M-AM axis, serve as conduits for localizing embryos to the AM pole. It is tempting to postulate that transverse folds could aid in spacing of embryos along the O-Cx axis in the scattering phase of embryo movement. However, despite aberrant longitudinal folds in *Wnt5a^cKO^* or *Rbpj^cKO^* mice pre-implantation, we did not observe embryo crowding. This suggests that longitudinal folds do not cause embryo crowding and conversely that transverse folds do not aid in fine embryo spacing.

### Transverse folds are naturally selected to avoid trapping of embryos in folds

Our data show that transverse folds resolve to form flat PIRs and the embryos eventually attach in the middle of a PIR. If the embryo requires a flat space to attach then the observation that the lumen forms transverse folds in the peri-implantation phase, instead of completely losing all its folds, is puzzling. An explanation can be inferred from the *Wnt5a^cKO^* and *Rbpj^cKO^* uteri, where the aberrant pre-implantation longitudinal folds do not completely resolve in the PIRs, causing embryo trapping at implantation sites. Hence, it is possible that luminal stretching in opposing directions can be induced only along the O-Cx axis and that luminal stretching can effectively flatten only transverse folds and not longitudinal folds. We did not observe a direct correlation between transverse folds and E-U alignment or chamber formation. Thus, we hypothesize that formation of transverse folds prior to implantation is an evolutionary selection that abolishes longitudinal folds that serve as potential traps for embryos and prevent them from localizing to the AM pole, thus disrupting implantation outcomes. This idea is further supported by the fact that even though the majority of pre-implantation folds in both *Wnt5a^cKO^* and *Rbpj^cKO^* uteri are predominantly longitudinal, less than half of the embryos are trapped in these aberrant folds and the remaining half escape the longitudinal folds and occupy flat regions at the AM pole. We conclude that, although the significance of transverse folds is still unclear, longitudinal folds are detrimental to embryo implantation and pregnancy success. A mouse model where the uterus completely lacks folds will help clarify the role of transverse folds during implantation and will be a subject of future studies.

### Peri-implantation region formation predefines implantation sites

In this study, we provide novel evidence that the implantation regions are formed by luminal patterning, after uterine sensing of embryos but before embryo spacing and arrival of embryos at implantation sites. As the embryo always attaches at the center of the flat PIR, in future, it will be important to identify chemotactic proteins that are specifically expressed in the central region of the flat PIR that attract the embryo to the potential implantation site.

Although it is well known that implantation in mice occurs at the AM pole, in women, implantation preferentially occurs in the fundus region of the uterus, near the posterior wall (Kim and Kim, 2017). PIR formation before embryo arrival at the implantation site could be key to successful implantation, even in monotocous species, independent of embryo spacing. Such knowledge can be useful in enhancing assisted reproductive technologies where the optimal stage and timing for embryo transfer during the window of implantation is still not understood (Enciso et al., 2021; Mackens et al., 2017).

### Chamber formation enables embryo rotation to orient ICM towards mesometrial pole

E-U alignment is crucial for a successful pregnancy. As the maternal uterine arteries enter the mouse uterus at the M pole, it is essential for the embryo to be oriented with the ICM facing the M pole. Although embryonic factors, such as FGFR2, have been shown to be crucial for blastocyst alignment within the chamber, the role of the uterine environment in E-U alignment is not yet known (Arman et al., 1998). Although it has been postulated that the ICM within the embryo is mobile and may migrate within the blastocoel to orient itself towards the M pole, there is no evidence to support this theory (Kirby et al., 1967). Embryo rotation as a whole, on the other hand, has been suggested to facilitate E-U alignment in several species, including horses and bats (Ginther, 1983; Rasweiler and Badwaik, 1999). In bats, the embryo rotates 90° after initiation of implantation, such that the ICM is oriented towards the maternal vasculature entering the uterus (Rasweiler and Badwaik, 1999). In horses, uterine contractions help in orienting the embryonic vesicle to ensure that the umbilical cord attaches to the allantoic sac. Embryo orientation in horses is further aided by cross ridging of endometrial folds (Ginther, 1998). In the mouse, several factors, including myometrial contractions, the physical shape of the chamber, the anchorage of trophoblast to the epithelium and the movement of uterine epithelial cells independently of the stroma have been proposed to aid with embryo rotation (Kirby et al., 1967). Although it has been suggested that the surface of the embryo has a uniform potential to initiate implantation and hence can attach at any random spot, our study shows that murine implantation always initiates at the mural trophectoderm opposite to the ICM. Furthermore, our data show that embryos are incapable of self-rotation, and that chamber formation and elongation are required for embryo rotation and to ensure E-U alignment. The fact that beads that lack any kind of internal axes form V-shaped chambers at the AM pole when coated with HBEGF (Yuan et al., 2018) suggests that that chamber formation occurs irrespective of the presence and, hence, the orientation of the embryo, but embryo orientation along the M-AM axis relies on appropriate chamber formation.

### Aberrant folding and resulting phenotypes in *Wnt5a^cKO^* and *Rbpj^cKO^* mice: comparison with published studies

It is well known that changes in luminal epithelial morphology during pregnancy are crucial for embryo implantation and pregnancy outcomes (Aplin and Ruane, 2017). By using the *Wnt5a^cKO^* mice as a model system for aberrant folds, we delineated the sequence of events that correlate how aberrant structure could cause embryo lethality (Table 1). Although Cha et al. (2014) showed that M-AM orientation of implantation sites in *Wnt5a^cKO^* uteri is abnormal at GD7, we show that embryo orientation defects arise due to unresolved longitudinal folds as early as GD4. Furthermore, Cha et al. showed 67% resorption rate at mid gestation and ∼50% reduction in litter size at birth in the *Wnt5a^cKO^* mice, our data show ∼14% resorption at GD6 (Table 1), ∼25% resorption at mid-gestation (Fig. S3B) and ∼35% reduction in litter size at birth (Table 1). The difference in penetrance of the embryo survival phenotypes could be due to the difference in background of mice used for these studies. Most importantly, our data support the observation that the proportion of embryos trapped in longitudinal folds at implantation (∼40%) correlates with the reduction in litter size (35%).

For the *Rbpj^cKO^* mice, Zhang et al. (2014) attributed E-U misalignment to abnormal luminal closure. However, our data suggest that the longitudinal folding pattern in the *Rbpj^cKO^* uteri is more likely to be the cause of E-U misalignment. This is further supported by the timing of luminal closure that happens around GD3 1600 h (Yoshinaga, 2013), whereas aberrations in pre-implantation fold formation are observed at GD3 1200 h in the *Rbpj^cKO^* uteri. Furthermore, while Zhang et al. concluded that defective luminal closure causes extra luminal folds in the *Rbpj^cKO^* uteri, we determined that luminal folds are present post-implantation in both control and *Rbpj^cKO^* uteri but, specifically, that the aberrant longitudinal folds in the *Rbpj^cKO^* mice disrupt embryo implantation.

### Transverse folding ensures E-U alignment for post-implantation embryo morphogenesis

The mechanical and structural aspects of the maternal environment that affect embryo morphogenesis are not fully understood (Matsuo and Hiramatsu, 2017). Mechanical forces exerted from the maternal tissue are required for egg cylinder morphogenesis, elongation of the embryo along the M-AM axis, correct specification of the distal visceral endoderm and anterior-posterior axis specification (Hiramatsu et al., 2013; Ueda et al., 2020). Our data show that aberrant luminal folding pattern in mice affects embryo orientation, which in turn affects buffer space between embryo and maternal decidua during morphogenesis. Future investigation is required to delineate how the abnormal space surrounding the embryo affects embryo morphogenesis potentially causing embryo mortality.

In summary, we have investigated how the 3D uterine luminal architecture affects implantation and continued pregnancy. We show that pre-implantation transverse folds along M-AM axis are essential for flat PIR formation, chamber formation and E-U alignment. Using two different mouse models that disrupt two independent signaling pathways, we show that a common phenotype of aberrant longitudinal folds disrupts chamber formation and E-U alignment, leading to compromised pregnancy outcomes. Further studies with mouse models that display disrupted uterine folding pattern can lead to a better understanding of the role of endometrial folds in pregnant women. Recurrent pregnancy loss is a prevalent disorder that affects pregnancy outcomes in women, but about 33% of the losses remain unexplained (Ford and Schust, 2009). In the future, research on uterine structure and folding can provide insights for diagnosis and treatment of unexplained pregnancy losses.

## MATERIALS AND METHODS

### Animals

CD1 mice and *Wnt5a^loxP/loxP^* (026626) (Ryu et al., 2013) mice were purchased from Charles River Laboratories and Jackson Labs, respectively. *Rbpj^cKO^* (*Pgr^Cre^ Rbpj^loxP/loxP^*) mice were generated as described previously (Strug et al., 2018) using the *Pgr^Cre^* mice (Soyal et al., 2005). *Wnt5a^cKO^* mice were generated by mating *Pgr^Cre^* mice with *Wnt5a^loxP/loxP^* mice. For pregnancy studies, adult females aged 6-8 weeks were mated with fertile males and the appearance of a vaginal plug was identified as gestational day (GD) 0.5. For time-course analysis, CD1 females were dissected between GD2 1200 h and GD4 1200 h. *Wnt5a^cKO^* and *Rbpj^cKO^* mice were dissected at GD3 1200 h, GD4 1200 h and/or 1800 h, and GD5 1200 h. All mouse studies were approved by the Michigan State University Institutional Animal Care and Use Committee.

### Whole-mount immunofluorescence

Whole-mount immunofluorescence was performed as previously described (Arora et al., 2016). Uteri were dissected from mice and fixed in DMSO:methanol (1:4). For immunostaining, uteri were rehydrated in methanol:PBT (1% Triton X-100 in PBS) (1:1) for 15 min, washed in PBT for 15 min and incubated in blocking solution (2% powdered milk in PBT) for 2 h at room temperature. Uteri were incubated with 1:500 concentration of primary antibodies diluted in blocking solution for five to seven nights at 4°C following which they were washed with 1% PBT four to six times for 30 min each at room temperature. Uteri were then incubated with secondary antibodies at 4°C for two or three nights, followed by four to six washes of 30 min each with 1% PBT and dehydration in methanol for 30 min. Uteri were then bleached in a solution of 3% H_2_O_2_ prepared in methanol overnight at 4°C. Finally, the samples were washed in 100% methanol for 30 min and cleared in BABB (1:2, benzyl alcohol:benzyl benzoate) (Sigma-Aldrich, 108006, B6630). Primary antibodies used included rat anti-CDH1 (M108, Takara Biosciences; 1:500), rabbit anti-FOXA2 (Abcam, ab108422; 1:500) and rabbit anti-PTGS2 (Abcam, ab16701; 1:500). Alexa Flour-conjugated secondary antibodies, donkey anti-rabbit 555 (A31572; 1:500) and goat anti-rat 647 (A21247; 1:500) were obtained from Invitrogen, and Hoechst (Sigma Aldrich, B2261) was used to stain the nucleus.

### Confocal microscopy

Samples were imaged using a Leica TCS SP8 X Confocal Laser Scanning Microscope System with white-light laser and 10× air objective. The entire length and thickness of the uterine horn was imaged using the tile scan function with *z* stacks 5-7 µm apart. Images were merged using Leica software LASX version 3.5.5.

### 3D reconstruction and image analysis

Image analysis was performed using commercial software Imaris v9.2.1 (Bitplane). The confocal image (.LIF) files were imported into the Surpass mode of Imaris. Using the channel arithmetics function, the FOXA2 signal of glands was subtracted from the epithelial CDH1 signal to obtain the lumen-only signal. The surface module of Imaris was then used to reconstruct the 3D surface of the lumen from the lumen-only channel. Similarly, for gland surfaces, the surface module was used to reconstruct the 3D surfaces from the FOXA2 channel. Embryo surfaces were reconstructed using the manual mode of the surface module from the Hoechst signal. Quantification of the luminal folding angle, the distance between folds, the distance of embryo from the middle of PIR, the embryo-uterine orientation, the space between the epiblast and the decidua, and the angle between the embryonic-abembryonic axis and the embryonic-PTGS2 axis was performed using the Measurement Points module in Imaris. For more details see the supplementary Materials and Methods.

### Statistics

Statistical analysis was performed using GraphPad Prism and Microsoft Excel. Student's unpaired *t*-test or Mann–Whitney *U*-test was used to compare two groups. One-way ANOVA or Kruskal–Wallis test was used to compare three or more groups. Dunn's multiple comparison test was used along with Kruskal–Wallis test when necessary. *P* values were adjusted for multiple comparisons. Pearson's correlation coefficient was used to test the correlation between angle of embryo alignment with uterine axis and ratio of space between embryo and decidua. *P*<0.05 was considered statistically significant.

## Supplementary Material

Supplementary information

Reviewer comments
