# Supplementary material for: Aberrant uterine folding in mice disrupts implantation chamber formation and alignment of embryo-uterine axes
Source: Development. 2022 Jun 13;149(11):dev200300. doi: 10.1242/dev.200300 (PMC9245188; doi:10.1242/dev.200300)
Supplement: Supplementary information [file develop-149-200300-s1.pdf]

Supplementary Methods:

Quantification of luminal folding angle: Each uterine horn was divided into 3 equal regions and a lumen surface of approximately 1000μm in length was reconstructed in each region. A MATLAB script for surface curvature (as previously described, (Arora et al., 2016)) was run on the lumen surfaces to determine the regions that are highly folded. A value of Cmean>0.15 was used as a threshold to **define a uterine fold**. Images of the 3D surfaces with curvature analysis, oriented along the M-AM axis, were captured using the snapshots module. The 2D images were re-imported into Imaris and visualized using the XY plane. Two orthogonal planes (XZ and YZ) were placed to define the M-AM and the anterior-posterior (A-P) axes respectively. The intersection of the M-AM and A-P planes was placed on the end of a fold. Using the Measurement Points module, the first point was placed at one end of the fold, followed by the second point at the other end of the fold where it meets the intersection of the M-AM and A-P planes, and finally the third point at the intersection of the M-AM plane and the plane of the 2D image (Fig. S1). The value of the angle was obtained using the statistics function and this angle **was called the fold angle**.

Quantification of distance between folds and gland numbers: The folds were visualized using an optical slicer using CDH1 or using a 3D Surface. A measurement point was placed on the surface of one fold and a second Measurement Point was placed on the adjacent fold. The distance between the points was obtained using the statistics function. All distances were normalized to the length of the uterine horn. To determine the number of glands between two folds, 3D surfaces of glands were reconstructed using the Surface module. The statistics function was applied on the gland surfaces to obtain the absolute number of glands. The number of glands between two folds per unit length was obtained by normalizing to the distance between the folds in the region of interest.

Quantification of distance of embryo from middle of PIR: The PIRs, folds and embryos were visualized using an optical slicer using CDH1, FOXA2 and Hoechst. The region between the first complete transverse fold running from M to AM pole at the anterior end of the PIR and the next complete transverse fold at the posterior end of the PIR was considered as the boundary of the PIR (Fig. S3). Partially resolved folds in the PIR that do not run all the way from the M pole to AM pole were not considered as a complete transverse fold. Using the Measurement Points module in Imaris, the first point (A) was placed on the base of a transverse fold on the anterior end of a PIR and second point (B) was placed on the base of the next transverse fold on the posterior end of the PIR. A third point (C) was placed on the embryo at the PIR or nearest to the PIR. Using the statistics function of Imaris, the Cartesian coordinates of the three points were obtained and

imported into Excel. The distance of C from the midpoint of line A-B was calculated to obtain the distance of embryo from the center of the PIR. The distances were normalized by dividing with the length of A-B.

Quantification of embryo-uterine orientation: The embryo at an implantation site on GD4 1800 h was visualized using an optical XY Orthogonal Slicer or Oblique Slicer (Fig. S10). An XZ Orthogonal Slicer was used to define the M-AM axis and was placed at the abembryonic pole of the embryo. Using the Measurement Points module, the first point was placed on the ICM on the M-AM plane. The second point was placed on the intersection of the M-AM plane and the abembryonic pole on the XY plane. The third point was placed on the intersection of the M-AM and XY planes. The value of the angle was obtained using the statistics function of Imaris.

Quantification of space between epiblast and maternal decidua: The embryo in a decidua was visualized along the dorsal-ventral plane using an XY Orthogonal Slicer or an Oblique Slicer. A YZ slicer indicating the A-P axis was placed on the embryo perpendicular to the M-AM plane. Using the Measurement Points module, the first length (x) on the anterior end of the embryo was measured by placing one point on intersection of the YZ plane and the maternal decidua, and the other point on the intersection of the epiblast and the YZ plane. Similarly, the second length (y) on the posterior end of the embryo was measured. The ratio of the lengths, z = maximum(x,y)/minimum(x,y).

Quantification of angle between embryonic-abembryonic axis and embryonic-PTGS2 axis: The surface module in Imaris was used to make a surface of the PTGS2 expression pattern around the implantation chamber and the center point (a) of the PTGS2 surface was obtained. The embryo at an implantation site was visualized using an optical XY Orthogonal Slicer or Oblique Slicer. Using the Measurement Points module, the first point was placed on point a. The second point was placed on the ICM (embryonic pole) (point b) and the third point was placed on the abembryonic pole (point c). The value of the angle between line ab and bc was obtained using the statistics function of Imaris.

Reference:

Arora, R., Fries, A., Oelerich, K., Marchuk, K., Sabeur, K., Giudice, L. C. and Laird, D. J. (2016). Insights from imaging the implanting embryo and the uterine environment in three-dimensions. *Development*, dev. 144386.

Development • Supplementary information

Supplementary figure 1.

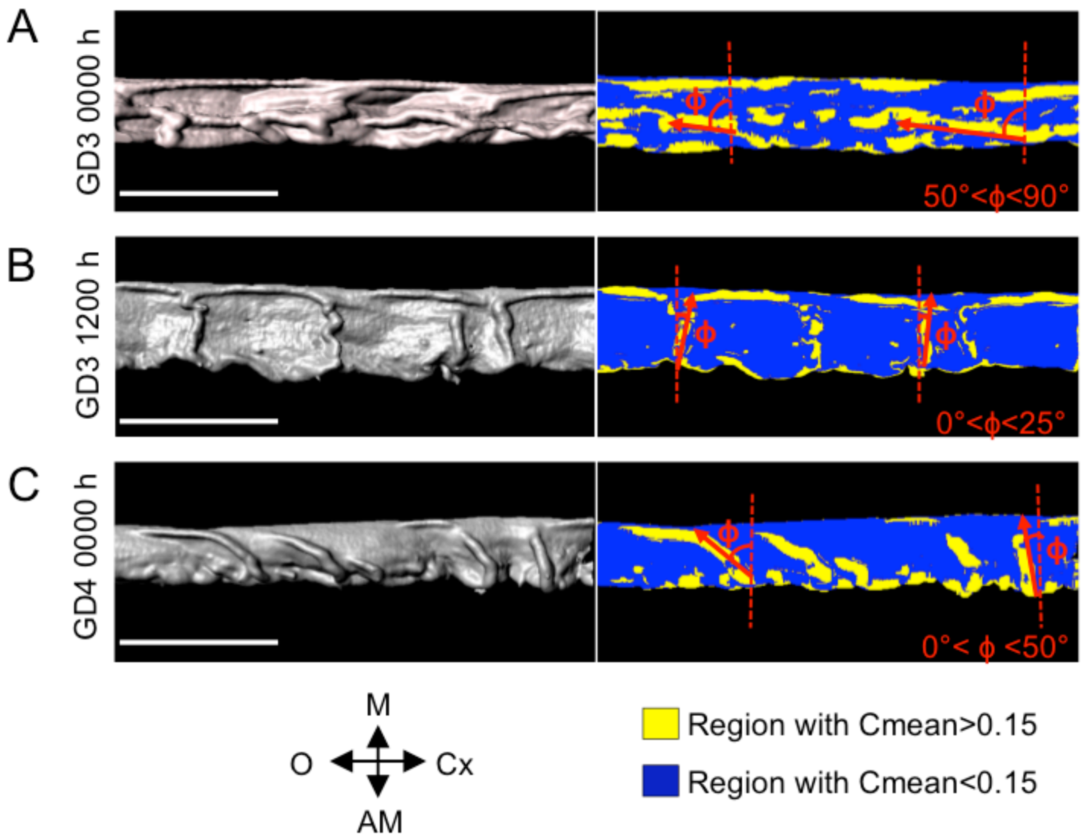

Fig. S1. Quantification of luminal fold angle with respect to M-AM axis
3D surface view and corresponding view with surface curvature analysis on GD3 0000 h (A), GD3 1200 h (B) and GD4 0000 h (C). Yellow arrows: transverse folds. Red arrows: longitudinal folds. Cmean – Curvature mean. ϕ - angle made by fold with respect to M-AM axis. (Scale bars: 1000μm).

Development • Supplementary information

Supplementary figure 2.

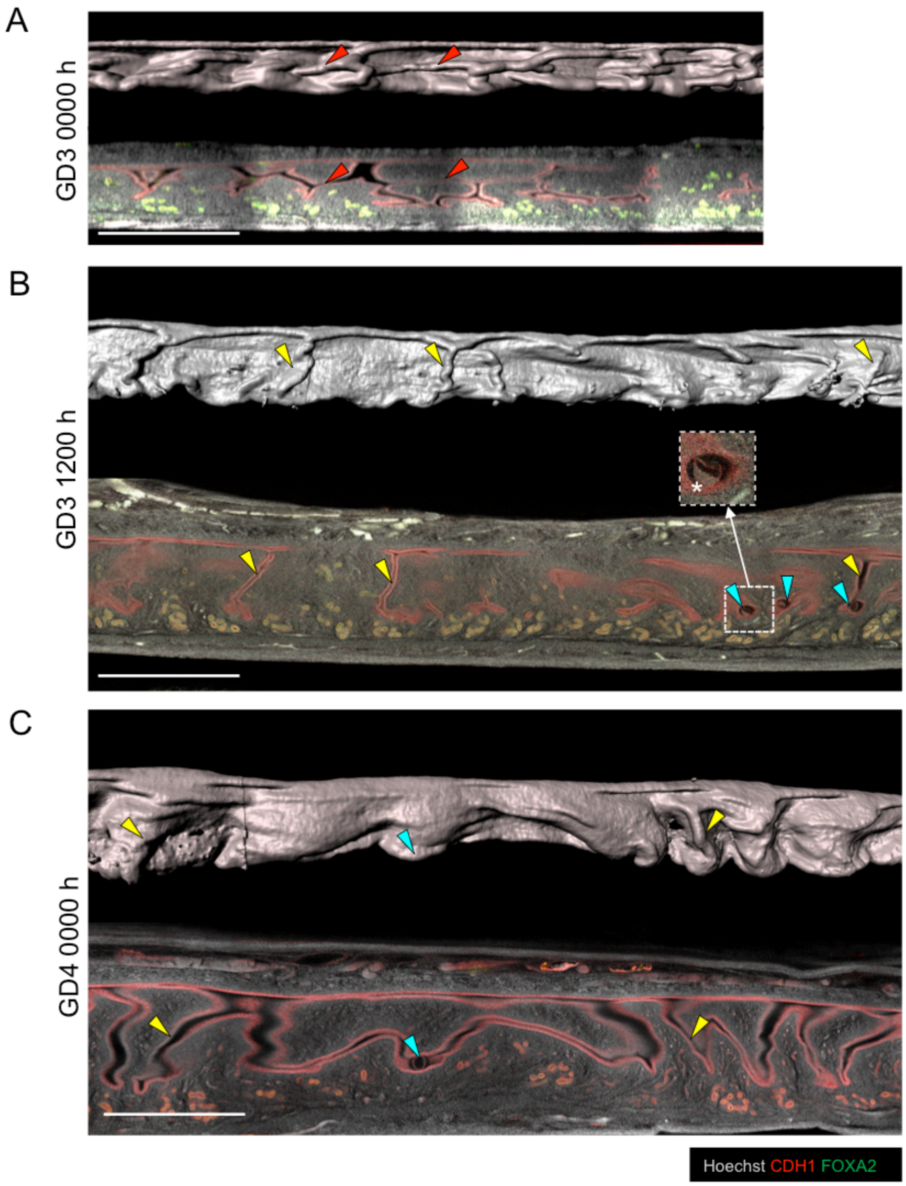

Fig. S2. 3D uterine folds (2D crypts) do not transform into implantation chambers. 3D surface view and corresponding optical slice view of uterine lumen on GD3 0000 h (A), GD3 1200 h (B) and GD4 0000 h (C). Yellow arrows: transverse folds. Red arrows: longitudinal folds. Blue arrows: embryos. Asterisk: inner cell mass. Dotted square shows optical slice view of intact embryo in the lumen. (Scale bars: 1000μm).

Development • Supplementary information

Supplementary figure 3.

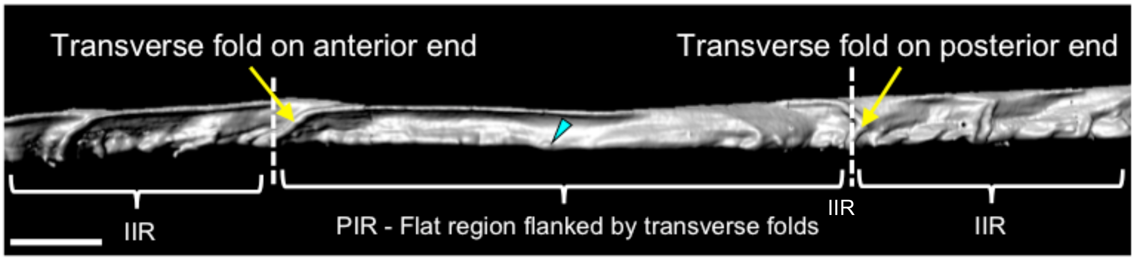

Fig. S3. Peri-implantation region boundary estimation.
3D surface view of lumen containing PIR on GD4 0000 h. Yellow arrows: transverse folds. PIR - peri-implantation region; IIR - inter-implantation region. (Scale bars: 500µm).

Supplementary figure 4.

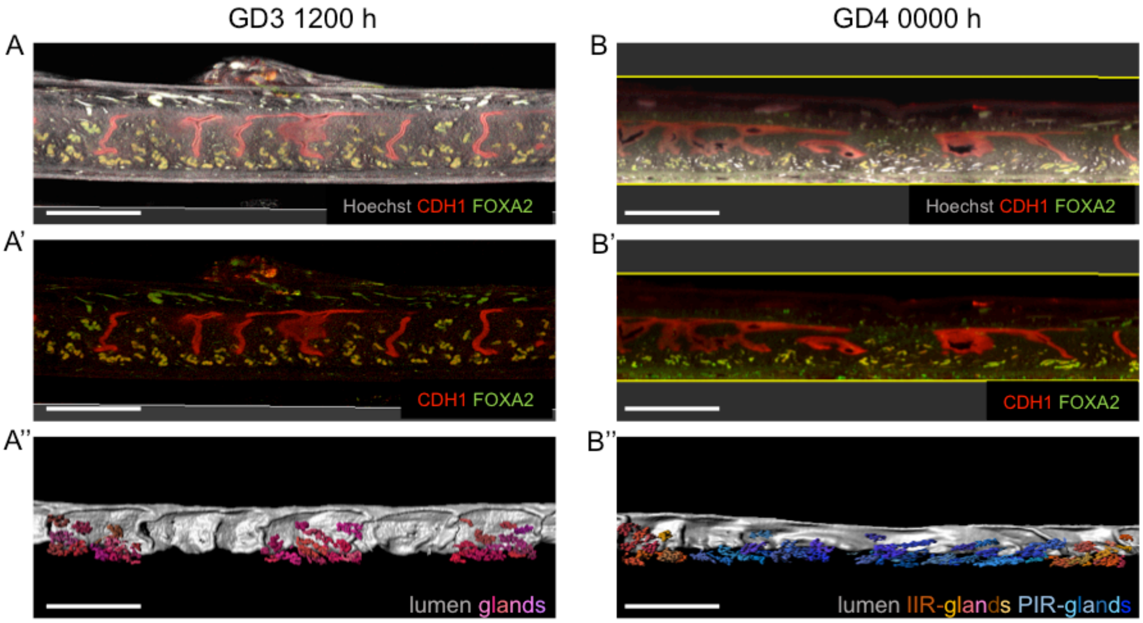

Fig. S4. Optical slice view and corresponding 3D view of surfaces in Fig 2A-B. Optical slice view (A,A') and 3D surface view (A'') at GD3 1200 h. Optical slice view (B,B') and 3D surface view (B'') at GD4 0000h. Gland surfaces are pseudo-colored to distinguish glands in IIR (orange) and PIR (blue). (Scale bars: 1000µm).

Development • Supplementary information

Supplementary figure 5.

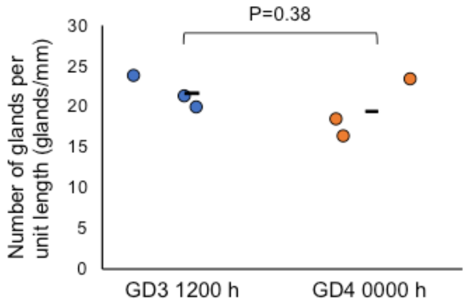

Fig. S5. Number of glands remain unchanged between GD3 and GD4. Quantification of number of glands per mm of horn length at GD3 1200 h (n=3) and GD4 0000 h (n=3). (P=0.38, Mann-Whitney U test). n - number of mice.

Supplementary figure 6.

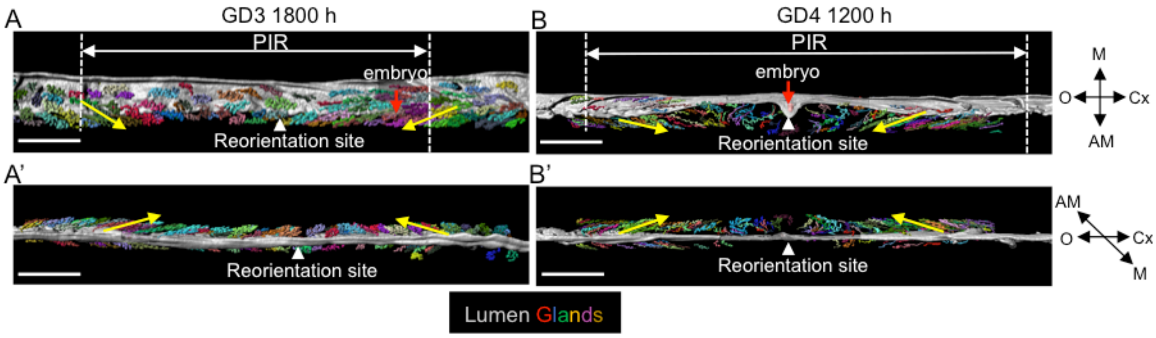

Fig. S6. Gland Reorientation precedes embryo arrival at implantation site. 3D surface view of lumen (gray) and glands (rainbow) around peri-implantation regions on GD3 1800 h (A,A’) and GD4 1200 h (B,B’). (A,B) View from ventral side; (A’B’) View from mesometrial side. White arrowhead indicates gland reorientation site. Yellow arrow indicates direction of gland reorientation. Red arrow indicates location of embryo. PIR - peri-implantation region. (Scale bars: 1000μm).

Development • Supplementary information

Supplementary figure 7.

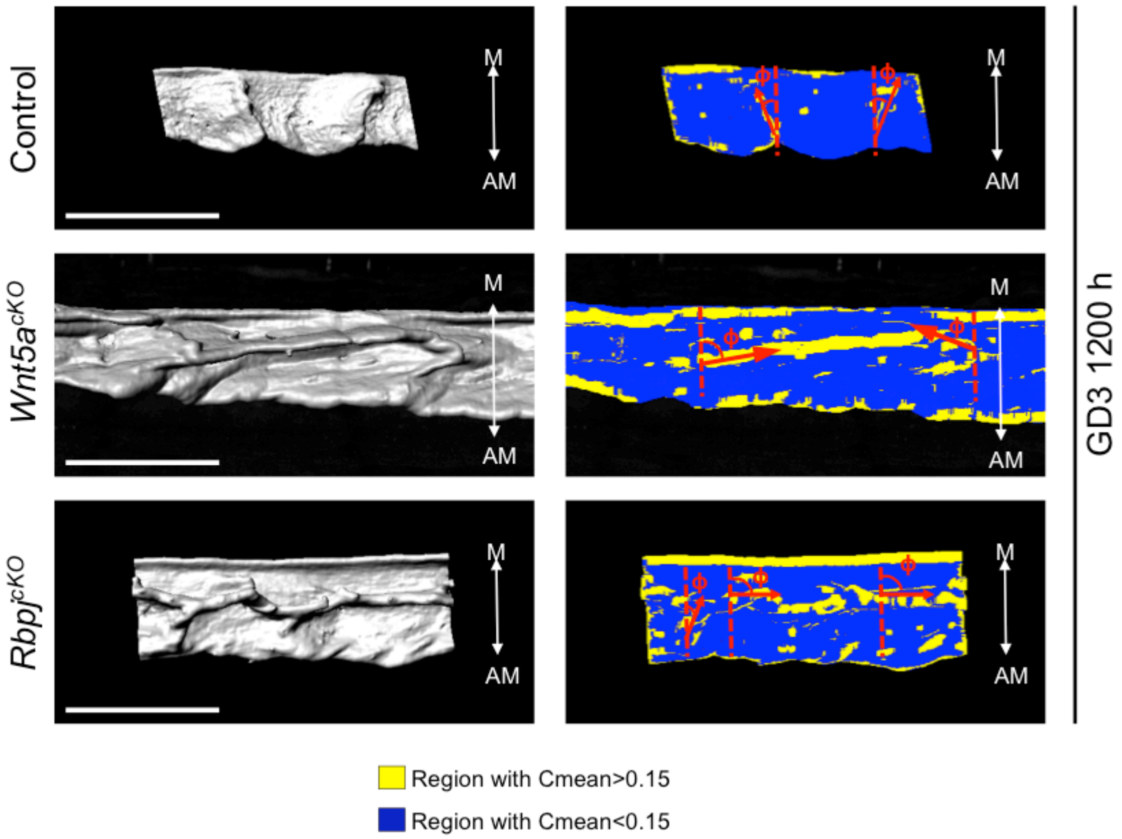

Fig. S7. Quantification of luminal fold angle in *Wnt5a*^{cKO} and *Rbpj*^{cKO}. 3D surface view and corresponding view with surface curvature analysis in control, *Wnt5a*^{cKO} and *Rbpj*^{cKO} at GD3 1200 h. Cmean - Curvature mean. ϕ - angle made by fold with respect to M-AM axis. (Scale bars: 1000μm).

Development • Supplementary information

Supplementary figure 8.

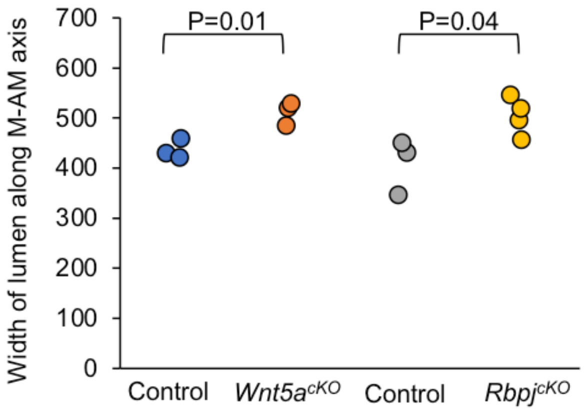

Fig. S8. Comparison of lumen widths in *Wnt5a*^{cKO} and *Rbpj*^{cKO} compared to controls. Quantification of width of lumen along M-AM axis in *Wnt5a*^{cKO} (n=3) and *Rbpj*^{cKO} (n=4) at GD3 1200 h along with respective controls (n=3 for each group). P, Student's unpaired t test.

Supplementary figure 9.

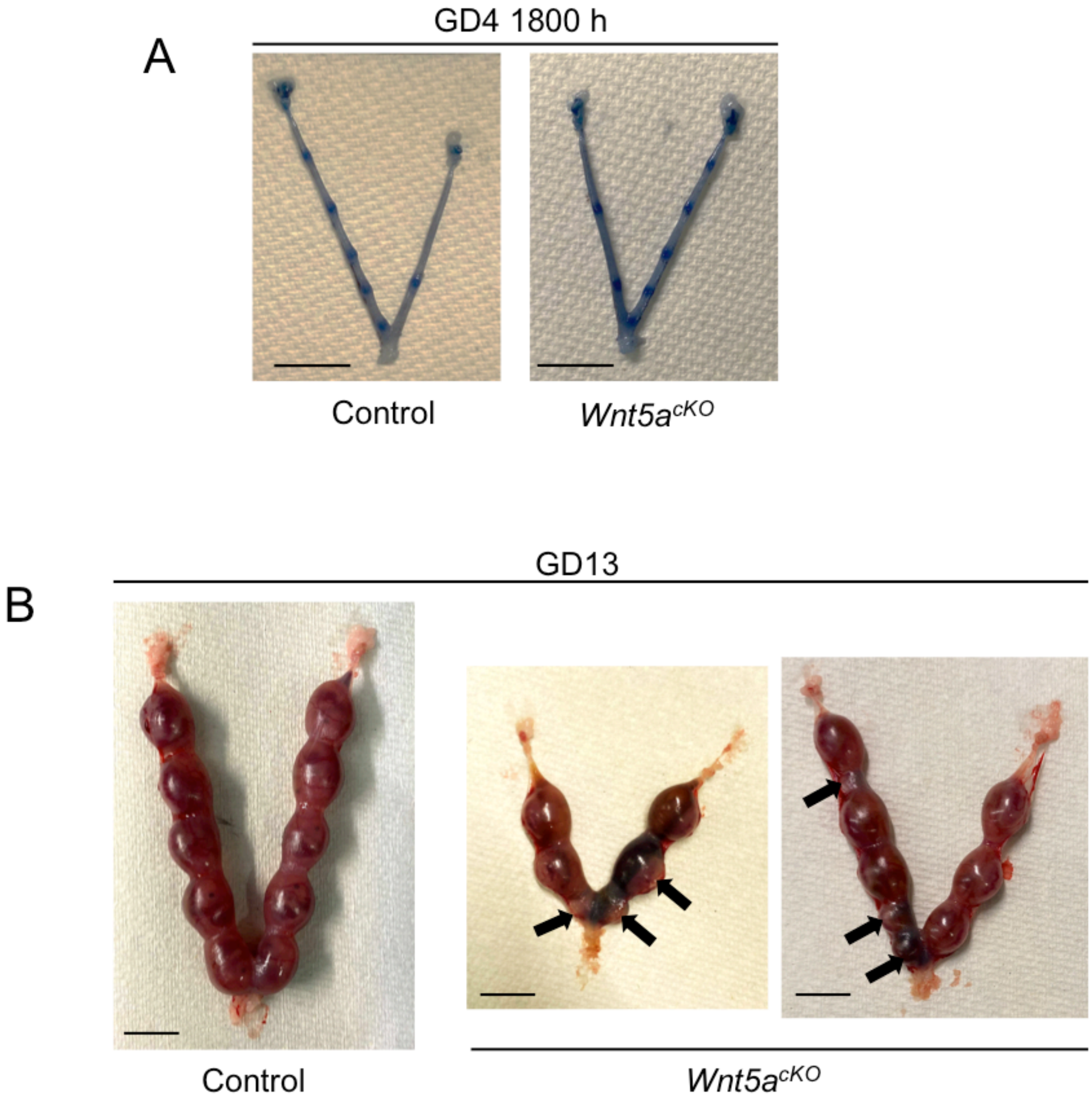

Fig. S9. Post-implantation embryo development in *Wnt5a*^{cKO} and control uteri

(A) Implantation sites in control and *Wnt5a*^{cKO} uteri on GD4 1800 h visualized using Evan's blue dye method. (B) GD13 implantation sites in control and *Wnt5a*^{cKO} uteri. Black arrows: resorption sites. (Scale bars: 1cm).

Development • Supplementary information

Supplementary figure 10.

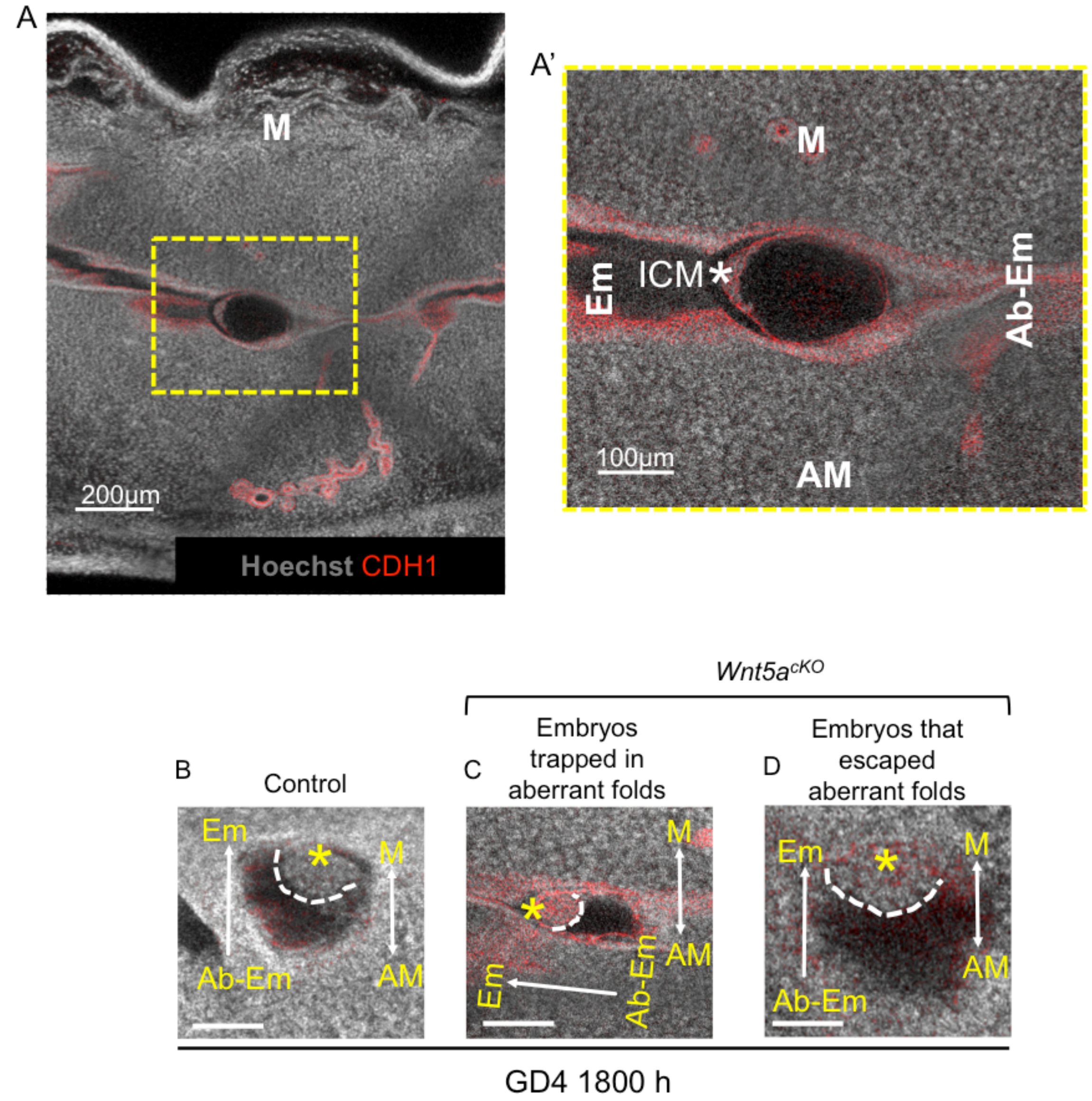

Fig. S10. High magnification images of embryo within the uterine lumen and determination of embryo axis.

(A,A’) Optical slice of embryo within the uterine lumen showing inner cell mass (ICM), embryonic pole (Em) and abembryonic pole (AbEm). (B-D) High magnification images of embryos in Fig. 4 G-I. Asterisk indicates inner cell mass. (Scale bars: 100µm).

Supplementary figure 11.

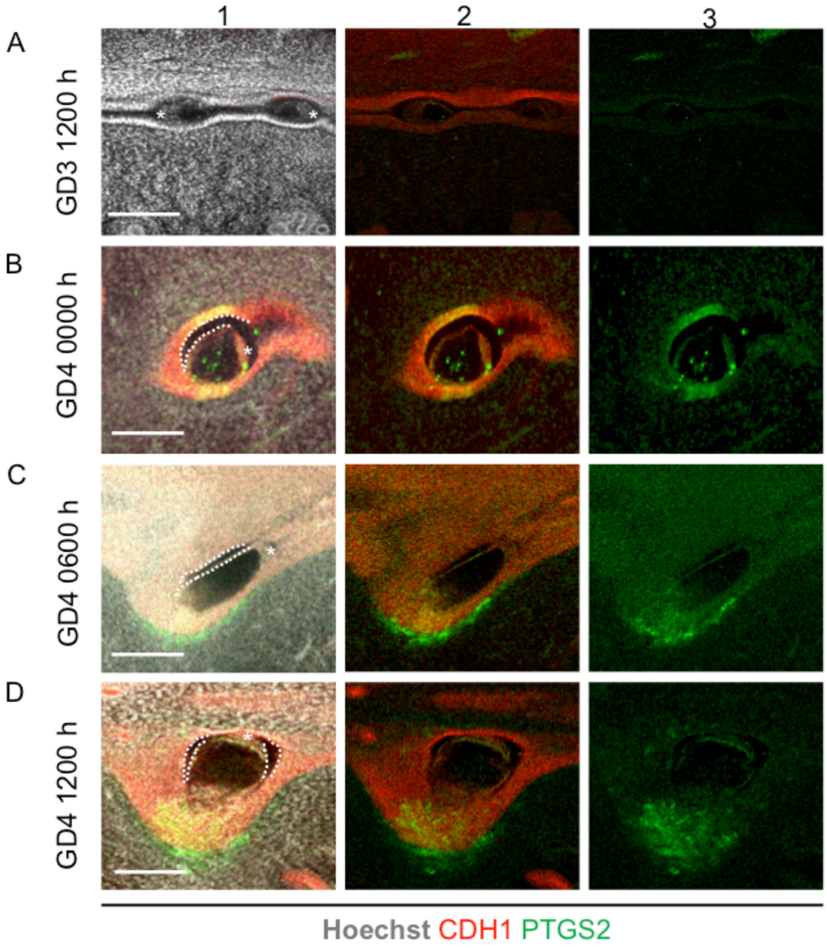

Fig. S11. High magnification images of PTGS2 expression during embryo alignment. Optical slice view of PTGS2 expression around embryos on GD3 1200 h (A), GD4 0000 h (B), GD4 0600 h (C) and GD4 1200 h (D). Panel 1: 2D optical slice with Hoechst, CDH1 and PTGS2. Panel 2: 2D optical slice with CDH1 and PTGS2. Panel 3: 2D optical slice with PTGS2 only. Asterisk indicates inner cell mass. (Scale bars: 200µm).

Development • Supplementary information

Supplementary figure 12.

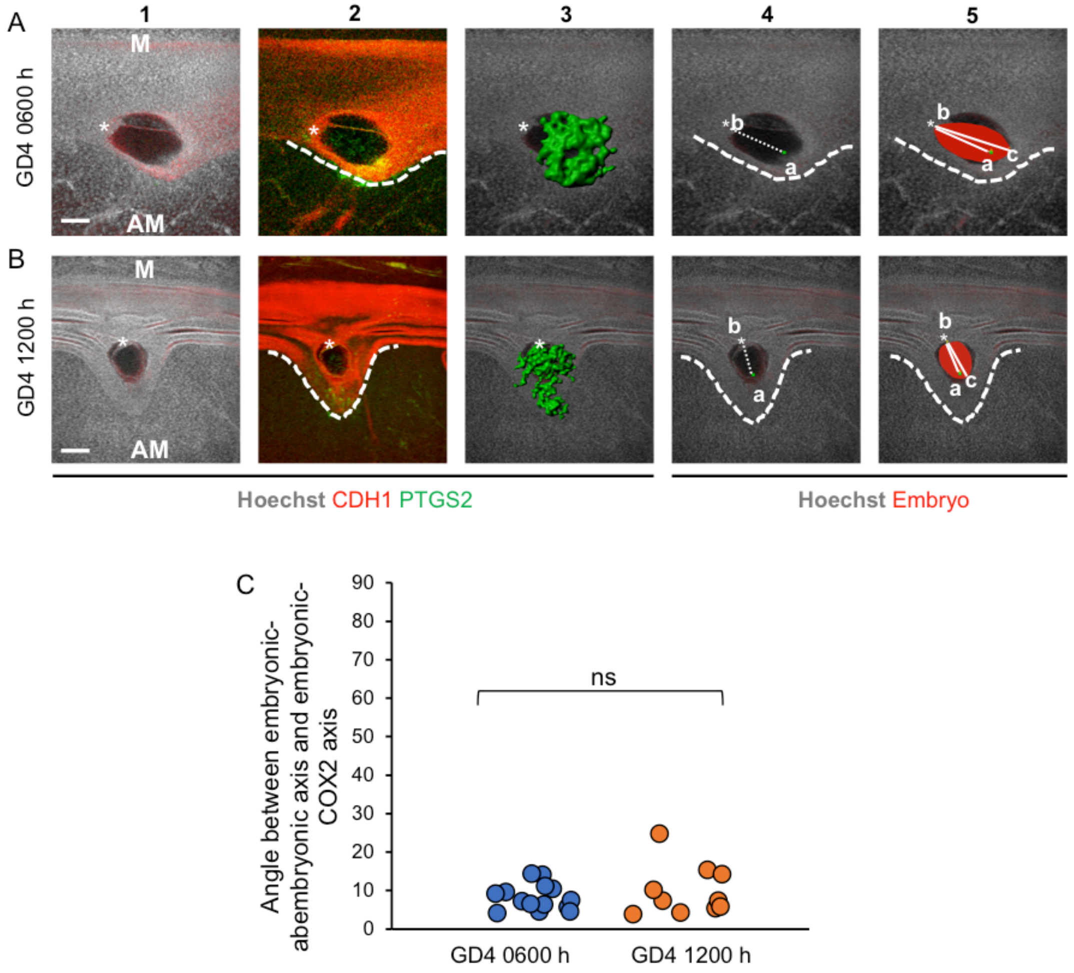

Fig. S12. Orientation of embryo relative to PTGS2 is maintained during chamber formation and elongation.

(A,B) Relationship between chamber formation, embryo orientation and PTGS2 expression in control mice on GD4 0600 h (A) and GD4 1200 h (B). Panel 1: 2D optical slice view of implantation site with Hoechst and CDH1. Panel 2: 2D optical slice view with PTGS2 (green) and CDH1 (red). Panel 3: optical slice with 3D surface of PTGS2 (green) around chamber. Panel 4: optical slice with embryonic-PTGS2 axis (line ab). Panel 5: optical slice with embryonic-PTGS2 axis and embryonic-abembryonic axis (line bc). White dotted line: implantation chamber. Asterisk: inner cell mass. (Scale bars: 100µm). (C) Quantification of angle between embryonic-PTGS2 axis and embryonic-abembryonic axis on GD4 0600 h (n=3, n_e=19) and GD4 1200 h (n=3, n_e=15). n – number of mice; n_e – number of embryos. (P=0.88, Mann-Whitney U test). ns - non-significant.

Development • Supplementary information

Supplementary figure 13.

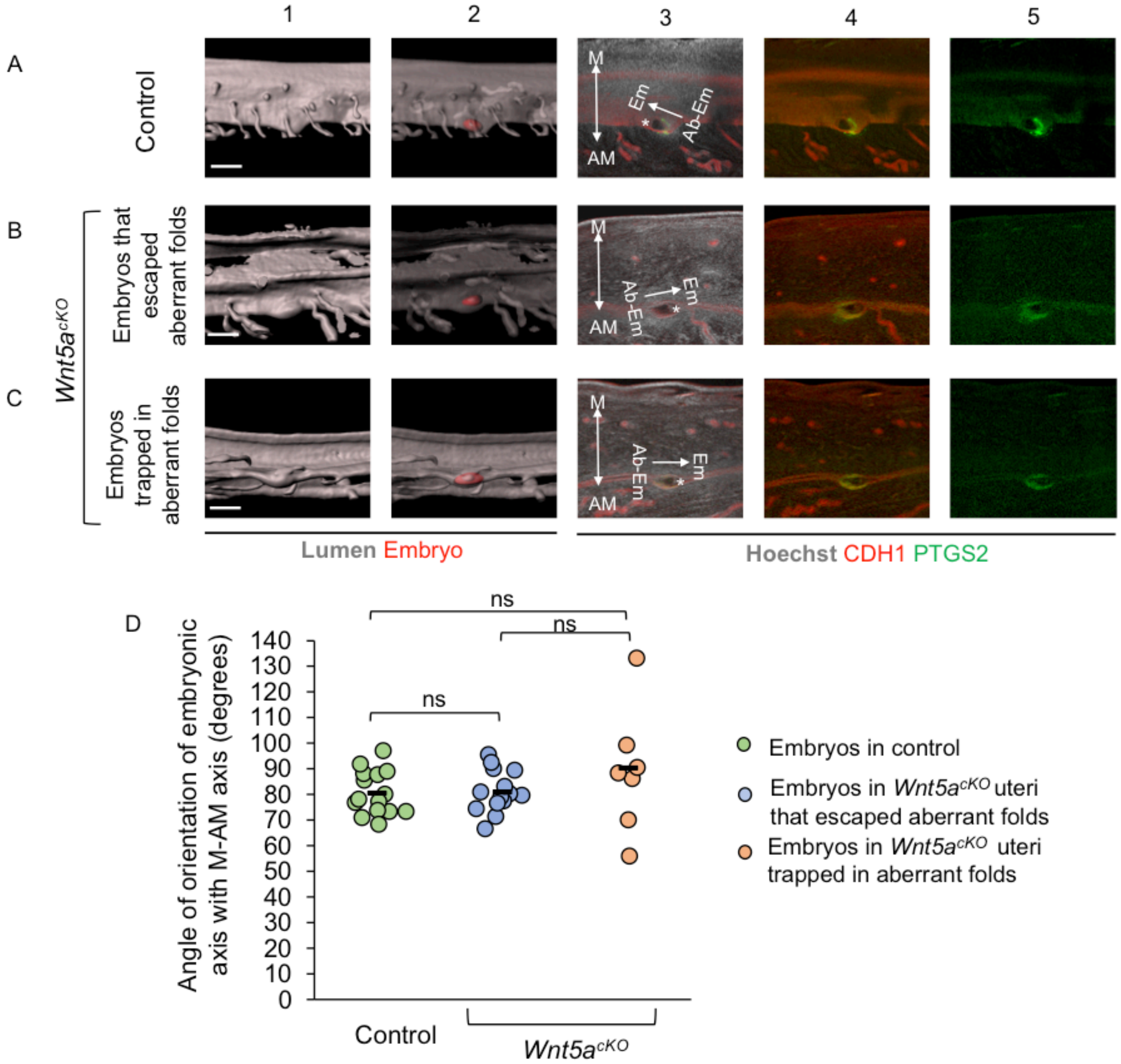

Fig. S13. Embryo-uterine alignment in *Wnt5a*^{cKO} at GD4 0000h.

(A-C) 3D surface view and optical slice view of implantation sites on GD4 1200 h in control (A) and *Wnt5a*^{cKO} (B,C) uteri. Implantation sites in *Wnt5a*^{cKO} uteri where embryos have escaped folds (B) or where embryos are trapped in folds (C). Panel 1: 3D lumen surface (gray). Panel 2: transparent 3D lumen and embryo (red) surface. Panel 3-5: optical slice view with Hoechst, CDH1 and PTGS2. (Scale bars: 200µm). Asterisk indicates inner cell mass. (D) Quantification of embryo orientation with respect to M-AM axis in control (n=3, n_e=21) and *Wnt5a*^{cKO} (n=6, n_e=15) uteri on GD4 1800 h. (P=0.54, Kruskal-Wallis test, Dunn's multiple comparison). ns - non-significant. n – number of mice; n_e – number of embryos.

Development • Supplementary information

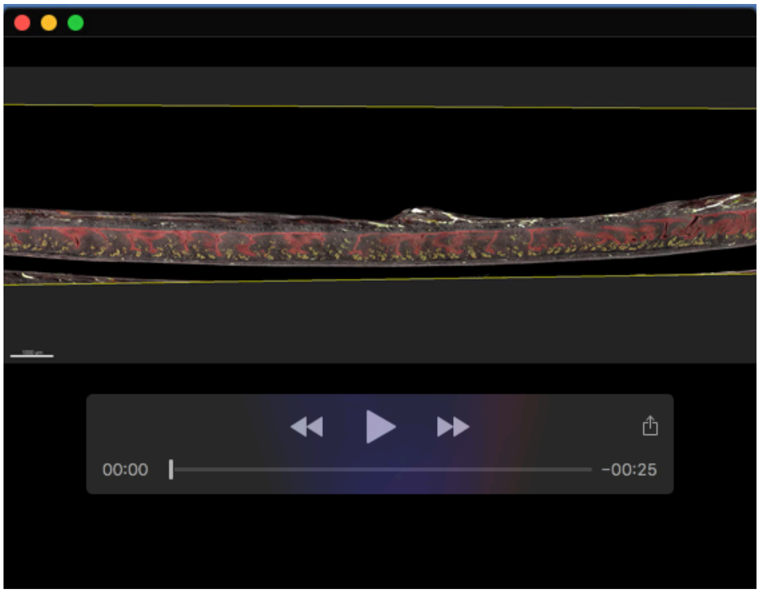

Movie 1. Visualization of transverse folds from multiple angles. Movie displays 2D optical slices and 3D surface view of the lumen in a GD3 1200 h wild type uterus. Optical slices display Hoechst (gray), CDH1 (red) and FOXA2 (green). In 3D surface view, gray surfaces indicate lumen and red surfaces indicate embryos within lumen

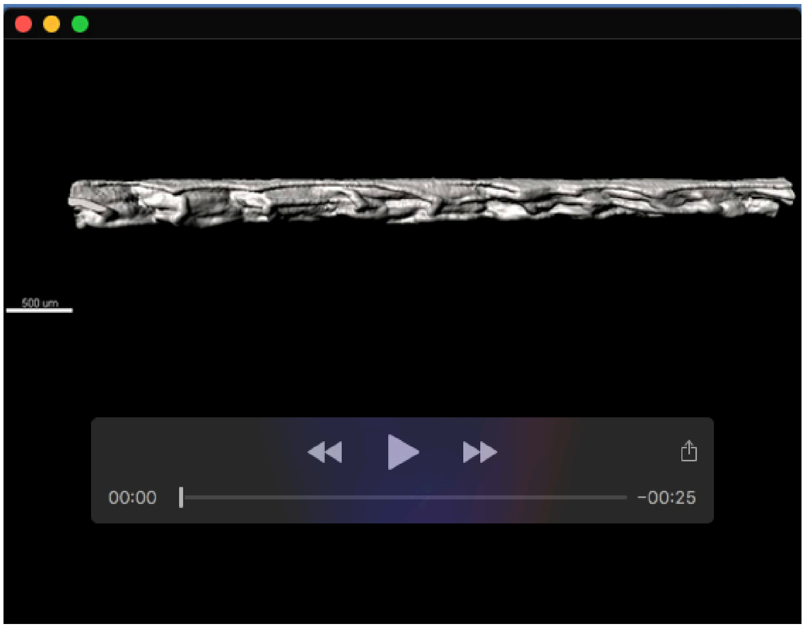

Movie 2. Surface curvature analysis of 3D lumen surface on GD3 0000 h. Movie displays 3D surface view of lumen in GD3 0000 h wild type uterus followed by surface curvature analysis highlighting the highly folded regions (yellow) compared to flat regions (blue). In 3D surface view, gray surfaces indicate lumen. Cmean>0.15 appears yellow and Cmean<0.15 appears blue. Cmean – Curvature mean.
