# Supplementary material for: Aberrant uterine folding in mice disrupts implantation chamber formation and alignment of embryo-uterine axes
Source: Development. 2022 Jun 13;149(11):dev200300. doi: 10.1242/dev.200300 (PMC9245188; doi:10.1242/dev.200300)
Supplement: Reviewer comments [file develop-149-200300_review_history.pdf]

Aberrant uterine folding in mice disrupts implantation chamber formation and alignment of embryo-uterine axes

Manoj K. Madhavan, Francesco J. DeMayo, John P. Lydon, Niraj R. Joshi, Asgerally T. Fazleabas and Ripla Arora

DOI: 10.1242/dev.200300

Editor: Patrick Tam

Review timeline

Original submission:	26 October 2021
Editorial decision:	24 November 2021
First revision received:	13 April 2022
Accepted:	3 May 2022

Original submission

First decision letter

MS ID#: DEVELOP/2021/200300

MS TITLE: Aberrant uterine folding in mice disrupts implantation chamber formation and embryo-uterine axes alignment

AUTHORS: Manoj Madhavan, Francesco J DeMayo, John P Lydon, Niraj R Joshi, Asgerally T Fazleabas, and Ripla Arora

I have now received all the referees' reports on the above manuscript, and have reached a decision. The referees' comments are appended below, or you can access them online: please go to BenchPress and click on the 'Manuscripts with Decisions' queue in the Author Area.

As you will see, the referees express considerable interest in your work, but have some criticisms and recommend a revision of your manuscript before we can consider publication. If you are able to revise the manuscript along the lines suggested, which may involve further work, I will be happy receive a revised version of the manuscript. Your revised paper will be re-reviewed by one or more of the original referees, and acceptance of your manuscript will depend on your addressing satisfactorily the reviewers' major concerns. Please also note that Development will normally permit only one round of major revision.

We are aware that you may be experiencing disruption to the normal running of your lab that make experimental revisions challenging. If it would be helpful, we encourage you to contact us to discuss your revision in greater detail. Please send us a point-by-point response indicating where you are able to address concerns raised (either experimentally or by changes to the text) and where you will not be able to do so within the normal timeframe of a revision. We will then provide further guidance. Please also note that we are happy to extend revision timeframes as necessary.

Please attend to all of the reviewers' comments and ensure that you clearly highlight all changes made in the revised manuscript. Please avoid using 'Tracked changes' in Word files as these are lost in PDF conversion. I should be grateful if you would also provide a point-by-point response detailing how you have dealt with the points raised by the reviewers in the 'Response to Reviewers' box. If you do not agree with any of their criticisms or suggestions please explain clearly why this is so.

© 2022. Published by The Company of Biologists under the terms of the Creative Commons Attribution License (https://creativecommons.org/licenses/by/4.0/).

Reviewer 1

Advance summary and potential significance to field

The authors identify uterine luminal folding dynamically changes along with uterine embryo location by utilizing high-resolution imaging and 3D-reconstruction techniques. In particular, they show that uterine transverse folds along the mesometrial-anti mesometrial axis are formed before spacing of embryos whereas implantation chambers form later at the implantation timing. Additionally, mice deficient in Wnt5a and Rbpj show aberrant longitudinal uterine folds, and embryos trapped by such longitudinal folds display incorrect embryo-uterine axes alignment. They also propose that these trapped and misaligned embryos are intimately linked to the resorption phenotype at later stages.

The luminal folding with embryo location they reveal here is very interesting and provide new insights into the implantation processes.

Comments for the author

Major concerns:
1)Roles of luminal folds are not completely verified. Transverse folds seem to be necessary for the movement of blastocysts toward the anti-mesometrial end, in which the implantation chamber is formed. However, without transverse folds, in Wnt5a mutant mother, blastocysts can move to the anti-mesometrial end and develop in the implantation chamber normally. Thus, the loss of longitudinal folds seems to be sufficient for correct implantation.
To address this issue, if possible, the authors had better use other types of mice which can form transverse but not longitudinal folds or can not form both types of folds.
2)Table 1:
It is still unclear that implanted blastocysts trapped by aberrant longitudinal folds directly correspond to the abnormal resorption phenotype in Wnt5a mutant mothers one-to-one. The author will be able to analyze misaligned embryos at GD7, GD9, GD11 stages and fill the gap of phenotypes between misalignment and resorption.
3)Figure 6:
The authors show that buffer space between the embryo and Reichert's membrane is asymmetrically produced in the Wnt5a mutant uterus. However, it is unclear whether Reichert's membrane is normally formed or not clearly shown. Immunohistochemistry of extracellular matrix molecules, markers for Reichert's membrane components, or ultrastructural analysis with TEM or SEM would be necessary. Asymmetric buffer space may not affect embryo morphogenesis if the buffer space itself can be formed.

Specific points:
i)The authors use the words “New and novel” several times in the discussion. What is the definition of new and novel? If they use these words, how do they demonstrate this is new or novel, otherwise, they had better remove such words.
ii) Description and percentage of Table 1 are difficult to understand. The total amount of percentages seems to be over 100. The total amount should be 100.
iii)Several sections of discussion appear to be too speculative such as human placental abnormalities which may not be directly related to rodent phenotypes.
In discussion: page 13 lines 22-27:
Sections regarding Cerberus1 expression and embryo-uterine alignment during implantation might be also too speculative.
iv) Figure 2A,2B legends:
There is no description regarding red and blue spots in the 3D images. What glands are stained?

Reviewer 2

Advance summary and potential significance to field

In the paper entitled “Aberrant uterine folding in mice disrupts implantation chamber formation and embryo-uterine axes alignment”, the authors describe morphological changes of mouse uterine

© 2022. Published by The Company of Biologists under the terms of the Creative Commons Attribution License (https://creativecommons.org/licenses/by/4.0/).

lumen during implantation periods. They also examined the relationship between the uterine M-AM axis and the embryonic axis in wild-type and the mutants for Wnt5a and Rbpj. They suggested that an aberrant folding pattern causes improper chamber formation and disruption of embryo-uterine axis alignment. This paper reports novel and important findings for the understanding of mouse implantation and embryonic development, especially morphological changes in the uterine environment for the embryonic development of the peri-implantation stage. However, I think the authors should provide more information for a better understanding of their observations, and some points should be clarified further. Their conclusions mentioned in the abstract are not fully supported directly by the experimental evidence as indicated following comments, and the manuscript should be revised accordingly.

Comments for the author

Major comments
1. Authors only show the projected images from one side of the uterus perpendicular to the M-AM axis, it is not easy to understand the 3D morphology of the uterus and folds. It would be nice if the authors provide images of the uterus from multiple angles for a better understanding of 3D morphology.
2. The definition of fold and fold angle is not clear. For example, some fold running parallel to the O-Cx axis in some regions also runs perpendicular to the O-Cx axis in other regions and these regions are connected. Views from multiple angles may also help to understand fold shape and the definition of fold angle.
3. It is not clear how the authors defined PIR shown in figure 1E-F. It would be nice if the authors clearly describe how the border between PIR and IIR are defined in these images?
4. Page5, the second paragraph. The authors mention that “These data support resolution of folds to form PIRs”. However, this interpretation is based on the assumption that the number of glands is stable during these stages. It might be possible that glands connecting to the luminal epithelium change their shape and numbers simultaneously with the changes in luminal epithelium. Can the authors show evidence that glands are not changed during these periods?
5. Page6, end of the second paragraph, they say “This suggests that the PIR is formed prior to embryo arrival at the implantation site”. But, as pointed in the former comment, the boundary between PIR and IIR is not clearly defined especially until GD3 1800h. And this may lead to variations in the distance of the embryo from the middle of the PIR shown in Fig. 4C. Is any other possible evidence to show that embryos move to the center of the PIR?
6. Orientation of the embryonic axis in the uterus is one of the important issues in the paper. But, the Em -AbEm axes of blastocysts indicated in Fig. 4 and Fig. 5 are not clear to me. It would be nice if the authors provide images of embryos with higher magnification so that readers can judge the embryonic axis from the images.
7. How is the orientation of the embryonic axis in the mutant mice when the implantation chamber is not formed?
8. In Fig, 5, Cox2 is shown as a potential decidualization marker, but the signals for COX2 immuno-staining are not clear in the images. It is shown that the initiation of COX2 in the LE is evident from GD4 0000h, but it is really difficult to see the signals. It would be better if the images of single-channel are also provided so that readers can recognize immuno-staining signals.
9. Please explain how the authors could judge if the embryo is in contact with the wall of the chamber on one side of the embryo as shown in the last line on page7? I wonder how they could define the attachment and the region of embryo attachment relative to the uterine epithelium.
10. The authors concluded that the embryo cannot rotate independently of the camber based on the observation of the contact of an embryo with the uterine wall. I do not think this conclusion is supported by any direct evidence, and it is necessary to show any direct evidence that the relative position of the embryo and uterine wall is maintained during axis alignment.
11. The overall morphology of the uterus in Wnt5a and Rbpj mutants is different from that of wild-type mice. And the question is how the authors applied the definition of fold morphology and angles. It would be nice if they explain in more detail.
12. In the first paragraph of the discussion on page 9, the authors summarized their study. I suggest some points be revised in the summary; “(1) uterine lumen forms transverse fold along the M-AM axis prior to implantation”. I think “exists” would be better than “forms”, because their observation started when folds already exist and they did not show any data without transverse fold before the implantation period. “(3) peri-implantation regions are pre-established by luminal patterning, prior to completion of embryo spacing”. I do not think they showed direct evidence to

© 2022. Published by The Company of Biologists under the terms of the Creative Commons Attribution License (https://creativecommons.org/licenses/by/4.0/).

support this. I also think there is no direct evidence to show a causal relationship suggesting “(5) chamber formation facilitates E-U alignment”.
13. Overall, the statistical information for each result is lacking.

Minor comments
1. It looks that the scale bars in Figures 1A-G, 2A-B, 3A-B, 4A-B, and 7A-B are not correct. Please check again and correct it properly.

Reviewer 3

Advance summary and potential significance to field

Following on from a recent paper from the group on distribution of embryos along the uterine corpus Using 3d imaging methods the authors show longitudinal channels or folds in the uterine lumen as well as lateral folds. They show changes with easy pregnancy and link the structures to embryo positioning, showing that flattening of specific regions precedes the initiation of formation of an implantation chamber, and that the nascent chamber orients the embryo correctly so that placentation can occur at the mesometrial side. They use 2 genetic models in which implantation failure occurs and reinterpret the observations in light of their new morphological insights.

This is another very interesting study, innovative and a clear step forward in understanding and I have no reason to delay its publication.

Comments for the author

It would help with clarity if in the legend to figure 1 the methodology for achieving the whole uterus luminal profile (ie Figure 1 A-G) was briefly summarised.
The gland staining is not shown independently, rather gland openings are marked (Fig 2). It would help if there was a Foxa2 image showing how the spacings of openings can be discerned.
I see no need to state that $P<0.05$ is taken as significant. They should simply cite the n numbers and the P values and leave readers to decide. This really only comes into play biologically in Figure 2 and I am not sure the differences claimed at one star are real. But please in the figures or legends give actual P values unless they are $<.001$
Typos page 3 paragraph 1 last word: unknown (not 'known')
page 6 para 3 line 3: bracket should move ... transverse)
page 6 line 2 from bottom: in or near

First revision

Author response to reviewers' comments

We thank the reviewers for their thoughtful comments and suggestions for clarification. Our responses are indicated below in blue and corresponding changes to the manuscript are highlighted in yellow.

Reviewer 1 Advance Summary and Potential Significance to Field:
The authors identify uterine luminal folding dynamically changes along with uterine embryo location by utilizing high-resolution imaging and 3D-reconstruction techniques. In particular, they show that uterine transverse folds along the mesometrial-anti mesometrial axis are formed before spacing of embryos whereas implantation chambers form later at the implantation timing. Additionally, mice deficient in Wnt5a and Rbpj show aberrant longitudinal uterine folds, and embryos trapped by such longitudinal folds display incorrect embryo-uterine axes alignment. They also propose that these trapped and misaligned embryos are intimately linked to the resorption phenotype at later stages.

© 2022. Published by The Company of Biologists under the terms of the Creative Commons Attribution License (https://creativecommons.org/licenses/by/4.0/).

The luminal folding with embryo location they reveal here is very interesting and provide new insights into the implantation processes.

Reviewer 1 Comments for the Author:
Major concerns:

1) Roles of luminal folds are not completely verified. Transverse folds seem to be necessary for the movement of blastocysts toward the anti-mesometrial end, in which the implantation chamber is formed. However, without transverse folds, in Wnt5a mutant mother, blastocysts can move to the anti-mesometrial end and develop in the implantation chamber normally. Thus, the loss of longitudinal folds seems to be sufficient for correct implantation.
To address this issue, if possible, the authors had better use other types of mice which can form transverse but not longitudinal folds or can not form both types of folds.
Response: We agree with the reviewer that this study suggests that loss of longitudinal folds is sufficient for the localization of embryos at the AM pole. However, despite a few attempts, we have not been able to find a mouse model which forms transverse folds only or cannot form any folds. The adult mouse uterus has random folds at any given stage of examination. The discussion has been modified to include this limitation in our current study.
Lines 385-394 reads "Thus, we hypothesize that formation of transverse folds prior to implantation, is an evolutionary selection to abolish longitudinal folds that serve as potential traps for embryos preventing them from localizing to the AM-pole thus disrupting implantation outcomes. This idea is further supported by the fact that even though majority of pre-implantation folds in both *Wnt5a*^{cKO} and *Rbpj*^{cKO} uteri are predominantly longitudinal, less than half of the embryos are trapped in these aberrant folds and the remaining half escape the longitudinal folds and occupy flat regions at the AM-pole. We conclude that although the significance of transverse folds is still unclear, longitudinal folds are detrimental to embryo implantation and pregnancy success. A mouse model where the uterus completely lacks folds will help clarify the role of transverse folds during implantation and will be a subject of future studies."

2) Table 1:
It is still unclear that implanted blastocysts trapped by aberrant longitudinal folds directly correspond to the abnormal resorption phenotype in Wnt5a mutant mothers one-to-one. The author will be able to analyze misaligned embryos at GD7, GD9, GD11 stages and fill the gap of phenotypes between misalignment and resorption.
Response: We thank the reviewer for this suggestion. However, our staining protocol and 3D imaging methodology is limited to GD6 for whole uterus and embryo analysis due to technical challenges of antibody penetration with increasing thickness of the decidua. In GD6 decidua using Hoechst staining we observe that ~14% of decidual sites in the *Wnt5a*^{cKO} are empty or have dying embryos as compared to 0% in controls (revised Table 1). Hence, we show that embryo resorption begins as early as GD6. It is possible that embryos trapped in folds die at different stages depending on the severity of the entrapment and misalignment.
In addition, using histological sections, it was previously shown that on GD7, *Wnt5a*^{cKO} have embryos that are misaligned with respect to the uterine axis (Cha et. al, 2014).

3) Figure 6:
The authors show that buffer space between the embryo and Reichert's membrane is asymmetrically produced in the Wnt5a mutant uterus. However, it is unclear whether Reichert's membrane is normally formed or not clearly shown. Immunohistochemistry of extracellular matrix molecules, markers for Reichert's membrane components, or ultrastructural analysis with TEM or SEM would be necessary. Asymmetric buffer space may not affect embryo morphogenesis if the buffer space itself can be formed.
Response:
We used Laminin-1 antibody staining to detect Reichert's membrane (RM) but unfortunately the antibody was not compatible with our whole-mount immunofluorescence staining protocol. Because of this we were unable to directly assess RM formation relative to embryo orientation. However, to detect RM we performed immunofluorescence staining of Laminin-1 on whole embryos dissected from *Wnt5a*^{cKO} and control uteri. At GD6 1200 h while the RM is nicely formed in 100% (n=7 embryos) of the embryos from control uteri and some (75%, n=9 embryos) embryos from the *Wnt5a*^{cKO} uteri, there is an absence of a functional RM in the smaller embryos from the *Wnt5a*^{cKO} uteri (25%, n=3 embryos).

© 2022. Published by The Company of Biologists under the terms of the Creative Commons Attribution License (https://creativecommons.org/licenses/by/4.0/).

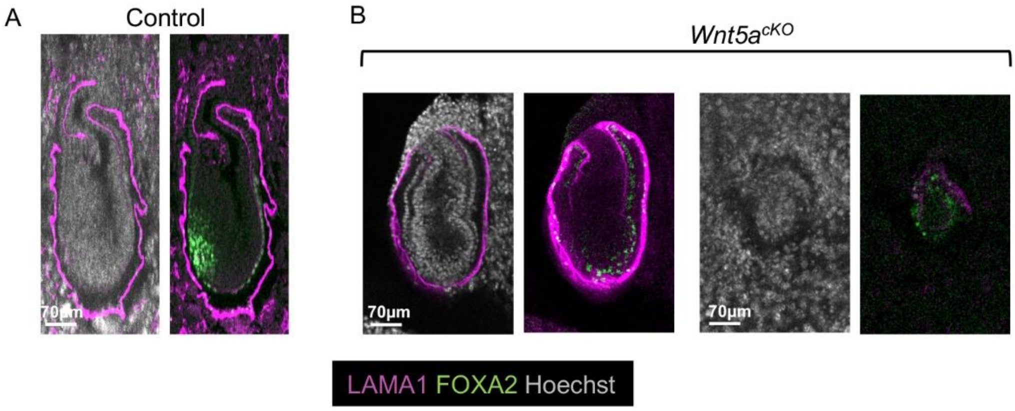

Reichert's membrane formation in *Wnt5a*^{cKO} and controls. Laminin subunit alpha 1 (LAMA1) expression in controls (A) and *Wnt5a*^{cKO} (B) at GD6 1200 h.

While these data point towards a defect in RM, it is unclear if the RM is defective due to poor alignment of the embryo or if the embryo is already dying and thus the RM is apoptosing. Due to these limitations, we have revised the results and discussions to remove any mention of the RM.

Further, as rightfully pointed out by the reviewer, on GD5 1200 h, using our whole tissue images, we are measuring the space between the embryo and the maternal decidua (not RM). Hence, we decided it would be more appropriate to use the term 'maternal tissue' instead of 'Reichert's membrane'. We have revised the text to substitute RM with maternal tissue and included a discussion about the role of maternal stiffness in specifying embryo axis (Hiramatsu et al., 2013). Lines 463-466 in discussion reads as "Mechanical forces exerted from the maternal tissue are required for egg cylinder morphogenesis, elongation of the embryo along the M-AM axis, correct specification of the distal visceral endoderm and anterior-posterior axis specification (Hiramatsu et al., 2013; Ueda et al., 2020)".

Specific points:
i) The authors use the words "New and novel" several times in the discussion. What is the definition of new and novel? If they use these words, how do they demonstrate this is new or novel, otherwise, they had better remove such words.
Response: The term new was used twice in the text to suggest possibility of discovery in the future, per the reviewer's suggestion we have modified these sentences to remove the term.
The term novel was used once in the discussion with respect to the discovery that implantation regions (IR) are formed by uterine patterning after embryo sensing. The idea that embryos can dictate formation of the implantation region in mouse as a species is a completely new idea thus we retain the use of the term novel in this context.

ii) Description and percentage of Table 1 are difficult to understand. The total amount of percentages seems to be over 100. The total amount should be 100.
Response: We thank the reviewer for the suggestion to modify the table. Table 1 has been updated to make it easier to understand. The table now highlights a correlation between the percentage of abnormal embryos at different stages of development and corresponding embryo loss in litter size.

iii) Several sections of discussion appear to be too speculative such as human placental abnormalities which may not be directly related to rodent phenotypes.
In discussion: page 13 lines 22-27:
Sections regarding Cerberus1 expression and embryo-uterine alignment during implantation might be also too speculative.
Response: We agree with the reviewer that the sections regarding Cerberus-1 and human placental abnormalities are speculative. We have removed this from the discussion.

iv) Figure 2A,2B legends:
There is no description regarding red and blue spots in the 3D images. What glands are stained?

© 2022. Published by The Company of Biologists under the terms of the Creative Commons Attribution License (https://creativecommons.org/licenses/by/4.0/).

Response: We thank the reviewer for pointing this out. All glands are stained with FOXA2 but to distinguish glands in the IIR and PIR they have been pseudocolored as red and blue. This information has now been included in the legend for Figure 2.

Reviewer 2 Advance Summary and Potential Significance to Field:
In the paper entitled “Aberrant uterine folding in mice disrupts implantation chamber formation and embryo-uterine axes alignment”, the authors describe morphological changes of mouse uterine lumen during implantation periods. They also examined the relationship between the uterine M-AM axis and the embryonic axis in wild-type and the mutants for Wnt5a and Rbpj. They suggested that an aberrant folding pattern causes improper chamber formation and disruption of embryo-uterine axis alignment. This paper reports novel and important findings for the understanding of mouse implantation and embryonic development, especially morphological changes in the uterine environment for the embryonic development of the peri-implantation stage. However, I think the authors should provide more information for a better understanding of their observations, and some points should be clarified further. Their conclusions mentioned in the abstract are not fully supported directly by the experimental evidence as indicated following comments, and the manuscript should be revised accordingly.

Reviewer 2 Comments for the Author:
Major comments

1. Authors only show the projected images from one side of the uterus perpendicular to the M-AM axis, it is not easy to understand the 3D morphology of the uterus and folds. It would be nice if the authors provide images of the uterus from multiple angles for a better understanding of 3D morphology.
Response: We thank the reviewer for this suggestion. For better understanding of the 3D morphology we have now included a Movie 1 that provides multiple views of the 3D surface of the lumen and folds.

2. The definition of fold and fold angle is not clear. For example, some fold running parallel to the O-Cx axis in some regions also runs perpendicular to the O-Cx axis in other regions and these regions are connected. Views from multiple angles may also help to understand fold shape and the definition of fold angle.
Response: The reviewer’s interpretation is correct that some folds running parallel may connect to others running perpendicular to the O-Cx axis. To make this more clear, we have now included Movie 2 that provides multiple views of regions containing longitudinal folds connected to transverse folds. In addition, we have clarified in the supplementary methods how folds and fold angles are defined.

3. It is not clear how the authors defined PIR shown in figure 1E-F. It would be nice if the authors clearly describe how the border between PIR and IIR are defined in these images?
Response: We thank the reviewer for this suggestion. To explain how the borders of the PIR are chosen for the analysis we have now included supplementary methods along with supplementary figure S3 (Fig. S3). Supplementary methods line 28 reads “The region between the first complete transverse fold running from M to AM pole at the anterior end of the PIR and the next complete transverse fold at the posterior end of the PIR was considered as the boundary of the PIR. Partially resolved folds in the PIR that do not run all the way from the M pole to AM pole were not considered as a complete transverse fold.”

4. Page5, the second paragraph. The authors mention that “These data support resolution of folds to form PIRs”. However, this interpretation is based on the assumption that the number of glands is stable during these stages. It might be possible that glands connecting to the luminal epithelium change their shape and numbers simultaneously with the changes in luminal epithelium. Can the authors show evidence that glands are not changed during these periods?
Response: In the mouse uterus GD3 is the receptive phase and is characterized by embryo movement and importantly an absence of proliferation in the epithelium deemed necessary for implantation. Epithelial proliferation occurs between GD0 and GD1 when estrogen levels are high. Starting GD2, as progesterone levels rise, there is a switch in proliferation from the epithelium to the stroma. Several studies have shown lack of epithelial proliferation on GD3 (Haraguchi et al., 2014; Hiraoka et al., 2020) (line 126).

© 2022. Published by The Company of Biologists under the terms of the Creative Commons Attribution License (https://creativecommons.org/licenses/by/4.0/).

Based on this literature, without proliferation on GD3, we do not think that the number of glands would change. In order to confirm this idea, we quantified the number of glands per unit length on GD3 1200 h and compared it to GD4 0000 h. Our quantification suggests no significant difference between the number of glands at these two time points (Fig. S5). These data support our claim that the number of glands remain unchanged between GD3 and GD4. It is true that there is a change in the shape of glands between GD3 and GD4 due to elongation of glands at the implantation site (Arora et. al, Development, 2016). But the change in shape of individual glands should not affect the number of glands branching off of the lumen and thus should not affect our analysis in Figure 2.

Lines 134-136 now reads "In addition, there is no significant difference between the total number of glands per unit length of horn on GD3 1200 h and GD4 0000h (PIRs and IIRs combined) (P=0.38, Mann-Whitney U test) (Fig. S5)"

5. Page6, end of the second paragraph, they say "This suggests that the PIR is formed prior to embryo arrival at the implantation site". But, as pointed in the former comment, the boundary between PIR and IIR is not clearly defined especially until GD3 1800h. And this may lead to variations in the distance of the embryo from the middle of the PIR shown in Fig. 4C. Is any other possible evidence to show that embryos move to the center of the PIR?
Response: We have included additional evidence for the movement of embryos towards the center of PIRs. We have previously shown that glands reorient towards the site of implantation on GD4 (Arora et al., 2016, Development). At GD3 1800 h, we find that gland reorientation has already occurred around the PIR and the embryo is away from the reorientation site. At GD4 1200h the glands remain oriented and the embryo has moved to the middle of the reoriented region. This information supports embryo movement towards the center of the PIR and has now been added to the results.
Lines 160-169 reads "Previously we have shown that the glands in the inter-implantation region reorient towards the implantation site on GD4 1200 h (Arora et al., 2016). The site of embryo implantation always coincides with the center of the gland reorientation site. Here, we show additional evidence that gland orientation occurs as early as GD3 1800 h when PIRs are first observed (Fig. S6A, A'). Interestingly, the center of gland reorientation site coincides with the center of PIRs at GD3 1800 h (Fig. S6A,A'). However, the embryos on GD3 1800 h are away from the gland reorientation sites, whereas at GD4 1200 h, implantation sites and the center of gland reorientation site and PIR coincides (Fig. S6B,B'). Hence, glands reorient towards the center of the PIRs even before embryos arrive at the potential implantation site. These data suggest that that the PIR and gland reoriented sites are formed prior to embryo arrival at the implantation site."

6. Orientation of the embryonic axis in the uterus is one of the important issues in the paper. But, the Em -AbEm axes of blastocysts indicated in Fig. 4 and Fig. 5 are not clear to me. It would be nice if the authors provide images of embryos with higher magnification so that readers can judge the embryonic axis from the images.
Response: We thank the reviewer for this feedback and have now included higher magnification images. Figure 4 has been modified to include higher magnification images of embryos in panels 4D-4F. Higher magnification images of Fig. 4G-I have been added to the supplementary figure 10 (Fig. S10) and we explain how the embryonic axis with respect to the uterine axis was defined. Higher magnification images of the embryos in Fig. 5 have been included in supplementary figure 11 (Fig. S11).

7. How is the orientation of the embryonic axis in the mutant mice when the implantation chamber is not formed?
Response: We thank for the reviewer for this excellent question. We observe that similar to controls, all embryos (including embryos trapped in folds) in *Wnt5a*^{cKO} uteri are aligned almost perpendicular to the M-AM axis when implantation is initiating but no implantation chamber is present. We have included supplementary figure 13 (Fig. S13) to show embryo axis alignment in *Wnt5a*^{cKO} at GD4 0000 h when implantation chamber formation has not initiated.
These new data have been included in Lines 253-260 as follows: "Based on these data, we hypothesized that embryos in the *Wnt5a*^{cKO} uteri should behave similar to embryos in control uteri until chamber formation initiates. Thus, we examined embryo alignment in *Wnt5a*^{cKO} uteri at GD4 0000 h when chambers are not yet formed. We observe that similar to controls (Fig. S13A), all embryos in the *Wnt5a*^{cKO} (including 33% of embryos trapped in longitudinal folds) are aligned with their Em-AbEm axis almost perpendicular to the M- AM axis irrespective of their localization (Fig.

© 2022. Published by The Company of Biologists under the terms of the Creative Commons Attribution License (https://creativecommons.org/licenses/by/4.0/).

S13B,C). There is no significant difference in the mean angle of alignment between embryos that escaped longitudinal folds (mean=81.09°) and embryos that are trapped in longitudinal folds (mean=88.91°) compared to controls (mean=80.64°) (P=0.54, Kruskal-Wallis test) (Fig. S13D). This suggests that in *Wnt5a*^{cKO}, the embryos that are trapped in longitudinal folds are unable to align with the M-AM axis, since they continue to be misaligned even at GD4 1800 h (Fig. 4H) whereas embryos that escape longitudinal folds are able to align with the M-AM axis at GD4 1800 h concomitant with chamber formation (Fig. 4I)."

8. In Fig, 5, Cox2 is shown as a potential decidualization marker, but the signals for COX2 immuno-staining are not clear in the images. It is shown that the initiation of COX2 in the LE is evident from GD4 0000h, but it is really difficult to see the signals. It would be better if the images of single-channel are also provided so that readers can recognize immuno-staining signals.
Response: We have included single channel images of COX2 in the Supplementary Figure 11 (Fig. S11)

9. Please explain how the authors could judge if the embryo is in contact with the wall of the chamber on one side of the embryo as shown in the last line on page7? I wonder how they could define the attachment and the region of embryo attachment relative to the uterine epithelium.
Response: We have included higher magnification images of embryos in Fig. 5 and in Fig. S11 to show that embryo is in contact with the wall of chamber. We also provide additional evidence regarding the relative position of the embryo and region of attachment being maintained during embryo rotation (Fig. S12).

COX2 (PTGS2) is expressed in the implantation chamber at the AM pole. We have shown that COX2 expression initiates in the LE, close to the mural TE (abembryonic pole) of the embryo, at GD4 1200 h. A few hours later on GD4 0600 h, COX2 shifts to the underlying stroma at the AM pole consistent with the initiation of implantation chamber formation. COX2 remains in the stroma at GD4 1200h when a functional implantation chamber is formed. Although the angle of alignment of the embryo changes from GD4 0600 h to GD4 1200h, COX2 expression is always observed in the stroma near the abembryonic pole of the embryo. We predicted that if the embryo is unable to rotate independent of the implantation chamber, COX2 expression pattern should always stay constant relative to the position of the embryonic-abembryonic axis. Hence, we measured the angle between the embryonic-abembryonic axis and the embryonic-COX2 axis on GD4 0600 h and GD4 1200 h (Fig S12 A and B). We observe that there is no significance difference in mean angles between the two time points which suggests that relative position of the embryo and COX2 is maintained during the entire period of embryo rotation (Fig. S12C). This data supports our claim that chamber formation rotates the embryo during implantation.

These changes are reflected in Lines 241-251 reads "We postulated that if embryo rotation is dependent on the implantation chamber then during the period when rotation is observed (between GD4 0600h and GD4 1200h), the position of the embryo with respect to the chamber should stay constant. To this end we used the ICM (for embryo position) and assessed its location with respect to the expression of stromal PTGS2 under the chamber (for chamber position). We measured the angle between the embryonic- abembryonic axis and the embryonic (ICM)-PTGS2 axis on GD4 0600 h and GD4 1200 h (Fig. S12A,B). We observe that there is no significance difference in the mean angle between these two axes at GD4 0600 h (mean=8.2°) and at GD4 1200 h (mean=9.8°). This suggests that the relative position of the embryo and stromal PTGS2 under the chamber is maintained during embryo rotation (P=0.88, Mann-Whitney U test) (Fig. S12C). These data suggest that embryo-uterine orientation during implantation is facilitated by formation of a chamber at the AM-pole in flat PIRs."

10. The authors concluded that the embryo cannot rotate independently of the camber based on the observation of the contact of an embryo with the uterine wall. I do not think this conclusion is supported by any direct evidence, and it is necessary to show any direct evidence that the relative position of the embryo and uterine wall is maintained during axis alignment.
Response: Please refer to response to comment 9.

11. The overall morphology of the uterus in Wnt5a and Rbpj mutants is different from that of wild-type mice. And the question is how the authors applied the definition of fold morphology and angles. It would be nice if they explain in more detail.

© 2022. Published by The Company of Biologists under the terms of the Creative Commons Attribution License (https://creativecommons.org/licenses/by/4.0/).

Response: The definition of folds and fold angles were consistently applied between controls, Wnt5a and Rbpj mutants to allow an unbiased comparison. Per the reviewer's suggestion we have now included a supplementary figure 7 (Fig. S7) that further demonstrates angle measurement in the *Wnt5a*^{cKO} and *Rbpj*^{cKO} uteri using surface curvature analysis.

12. In the first paragraph of the discussion on page 9, the authors summarized their study. I suggest some points be revised in the summary; "(1) uterine lumen forms transverse fold along the M-AM axis prior to implantation". I think "exists" would be better than "forms", because their observation started when folds already exist and they did not show any data without transverse fold before the implantation period. "(3) peri- implantation regions are pre-established by luminal patterning, prior to completion of embryo spacing". I do not think they showed direct evidence to support this. I also think there is no direct evidence to show a causal relationship suggesting "(5) chamber formation facilitates E-U alignment".

Response: For (1), we show that uterine lumen is randomly folded on GD2 1200 h. During embryo movement between GD3 0000 h and GD3 0600 h, the lumen forms longitudinal folds and during embryo spacing at GD3 1200 h, it forms transverse folds. We have now modified the statement and now lines 311-313 reads "(1) randomly folded uterine lumen organizes into longitudinal folds during unidirectional embryo movement and later into transverse folds during embryo spacing prior to implantation"

For (3), we have shown additional evidence that peri-implantation regions are formed prior to embryo spacing. Please refer to comment 5 and Fig. S6. Hence, we have not modified point (3).

For (5), we have again shown additional evidence for the rotation of embryo by the implantation chamber (Fig. S12). Please refer to response for comments 9 and 10.

13. Overall, the statistical information for each result is lacking.
Response: We have included detailed statistical information (P values and type of test performed) for each result in the figure legends and where possible in the main text.
Due to word limit constraints we have not repeated the kind of statistical tests performed in the results because this is part of the figure legends.

Minor comments
1.It looks that the scale bars in Figures 1A-G, 2A-B, 3A-B, 4A-B, and 7A-B are not correct. Please check again and correct it properly.
Response: All the scale bars have been verified. Please note that the scale bars in Figures 1E and 1G are smaller compared to Figures 1A-D and 1F. The latter images have been zoomed out to accommodate two implantation regions.
Per the reviewer's suggestion we reanalyzed the scale bars in Figures 4 and 7. We noted that the width of the lumens in *Wnt5a*^{cKO} and *Rbpj*^{cKO} is significantly larger compared to the respective controls. This leads to the appearance that the scale bars are not accurate. We have now included supplementary figure S8 (Fig. S8) and a note in the results section highlighting that the width of the uterine lumen along the M-AM axis is larger in the *Wnt5a*^{cKO} and *Rbpj*^{cKO} uteri compared to control uteri.

Reviewer 3 Advance Summary and Potential Significance to Field:
Following on from a recent paper from the group on distribution of embryos along the uterine corpus Using 3d imaging methods the authors show longitudinal channels or folds in the uterine lumen as well as lateral folds. They show changes with easy pregnancy and link the structures to embryo positioning, showing that flattening of specific regions precedes the initiation of formation of an implantation chamber, and that the nascent chamber orients the embryo correctly so that placentation can occur at the mesometrial side. They use 2 genetic models in which implantation failure occurs and reinterpret the observations in light of their new morphological insights.

This is another very interesting study, innovative and a clear step forward in understanding and I have no reason to delay its publication.
Response: We thank the reviewer for the positive comments.

© 2022. Published by The Company of Biologists under the terms of the Creative Commons Attribution License (https://creativecommons.org/licenses/by/4.0/).

Reviewer 3 Comments for the Author:
It would help with clarity if in the legend to figure 1 the methodology for achieving the whole uterus luminal profile (ie Figure 1 A-G) was briefly summarised.
Response: The methodology for obtaining the 3D lumen surfaces is summarized in the materials and methods (Lines 518-520). Due to limitation on number words we have not repeated the methodology in the figure legends.

The gland staining is not shown independently, rather gland openings are marked (Fig 2). It would help if there was a Foxa2 image showing how the spacings of openings can be discerned.
Response: We have included FOXA2 staining of images from Fig. 2 in Supplementary figure S4 (Fig. S4).

I see no need to state that $P<0.05$ is taken as significant. They should simply cite the n numbers and the P values and leave readers to decide. This really only comes into play biologically in Figure 2 and I am not sure the differences claimed at one star are real.
But please in the figures or legends give actual P values unless they are <.001. Response: Per the reviewer's suggestion we have now included the P values in the main text and figures wherever $P<0.05$.

Typos
page 3 paragraph 1 last word: unknown (not 'known') Response: This has been edited as suggested.

page 6 para 3 line 3: bracket should move ... transverse) Response: This has been edited as suggested.

page 6 line 2 from bottom: in or near Response: This has been edited as suggested.

Note to reviewers: We have also replaced COX2 with PTGS2 and ECAD with CDH1 to comply with MGI nomenclature guidelines.

Second decision letter

MS ID#: DEVELOP/2021/200300

MS TITLE: Aberrant uterine folding in mice disrupts implantation chamber formation and embryo-uterine axes alignment

AUTHORS: Manoj Madhavan, Francesco J DeMayo, John P Lydon, Niraj R Joshi, Asgerally T Fazleabas, and Ripla Arora
ARTICLE TYPE: Research Article

I am happy to tell you that your manuscript has been accepted for publication in Development, pending our standard ethics checks.

Reviewer 1

Advance summary and potential significance to field

The authors identify uterine luminal folding dynamically changes along with uterine embryo location by utilizing high-resolution imaging and 3D-reconstruction techniques. In particular, they show that uterine transverse folds along the mesometrial-anti mesometrial axis are formed before the spacing of embryos whereas implantation chambers form later at the implantation timing. Additionally, mice deficient in Wnt5a and Rbpj show aberrant longitudinal uterine folds, and embryos trapped by such longitudinal folds display incorrect embryo-uterine axes alignment. They also propose that these trapped and misaligned embryos are intimately linked to the resorption

© 2022. Published by The Company of Biologists under the terms of the Creative Commons Attribution License (https://creativecommons.org/licenses/by/4.0/).

phenotype at later stages. The luminal folding with embryo location they reveal here is very interesting and provides new insights into the implantation processes.

Comments for the author

Madhavan et al. have addressed many of the points I requested in the first review clearly and improved the manuscript substantially. So, I will support the publication.

Reviewer 2

Advance summary and potential significance to field

In this paper, the authors describe morphological changes in mouse uterine lumen during implantation periods. They also examined the relationship between the uterine M-AM axis and the embryonic axis in wild-type and the mutants for Wnt5a and Rbpj. They suggest that the implantation chamber facilitates embryo rotation to align embryonic axes along the uterine mesometrial-antimesometrial axis. This paper reports novel and important findings for the understanding of mouse implantation and embryonic development.

Comments for the author

The authors have satisfactorily addressed most of my concerns.

Reviewer 3

Advance summary and potential significance to field

The ms is improved as a result of the revisions.

Comments for the author

n/a

© 2022. Published by The Company of Biologists under the terms of the Creative Commons Attribution License (https://creativecommons.org/licenses/by/4.0/).
